# Safety and immunogenicity of a ChAdOx1 vaccine against Rift Valley Fever in adults: an open-label, non-randomised, first-in-human phase 1 clinical trial

**DOI:** 10.1016/S1473-3099(23)00068-3

**Published:** 2023-04-13

**Authors:** Daniel Jenkin, Daniel Wright, Pedro M. Folegatti, Abigail Platt, Ian Poulton, Alison Lawrie, Nguyen Tran, Amy Boyd, Cheryl Turner, John N. Gitonga, Henry K. Karanja, Daisy Mugo, Katie J. Ewer, Thomas A. Bowden, Sarah C. Gilbert, Bryan Charleston, Pontiano Kaleebu, Adrian V. S. Hill, George M. Warimwe

**Affiliations:** 1 The Jenner Institute, University of Oxford, UK; 2 Department of Paediatrics, University of Oxford, UK; 3 KEMRI-Wellcome Trust Research Programme, Kenya; 4 Wellcome Centre for Human Genetics, Division of Structural Biology, University of Oxford, UK; 5 Pandemic Sciences Institute, University of Oxford, Oxford, UK; 6 Chinese Academy of Medical Science (CAMS) Oxford Institute (COI), University of Oxford; Oxford, UK; 7 The Pirbright Institute, UK; 8 Uganda Virus Research Institute, Entebbe, Uganda; 9 MRC/UVRI & LSHTM Uganda Research Unit, Entebbe, Uganda; 10 Centre for Tropical Medicine and Global Health, University of Oxford, UK

## Abstract

**Background:**

Rift Valley Fever (RVF) is a viral epidemic illness prevalent in Africa that can be fatal or result in debilitating sequelae in humans. No vaccines are available for human use. We evaluated the safety and immunogenicity of a non-replicating simian adenovirus (ChAdOx1)-vectored RVF vaccine in humans.

**Methods:**

We conducted a phase 1 first-in-human open-label dose-escalation trial in healthy adults in the United Kingdom aged 18 to 55 years (NCT04754776). Participants were non-randomly allocated to receive a single ChAdOx1 RVF vaccination at a dose of either 5×10^9^ virus particles (vp), 2·5×10^10^ vp or 5×10^10^ vp and were followed up for 3 months. The main study objectives were safety and immunogenicity. Primary outcome measures were assessment of adverse events and secondary outcome measures were RVF neutralising antibody titres, RVF GnGc binding antibody titres (ELISA) and cellular response (ELISpot). All participants that received a vaccine were included in analyses.

**Findings:**

Between 11^th^ June 2021 and 13^th^ January 2022, 15 volunteers received a single dose of either 5×10^9^ vp (n=3), 2·5×10^10^ vp (n=6) or 5×10^10^ vp (n=6) ChAdOx1 RVF. ChAdOx1 RVF was well tolerated with no serious adverse events. Adverse events were short lived and predominantly mild. RVF neutralising antibodies (nAb) were detectable across all dose groups, with all vaccinees in the 5×10^10^ vp dose group mounting high nAb titres that peaked at day 28 post-vaccination and persisted through the 3 month follow up period. High titres of binding IgG targeting Gc glycoprotein were detected while those targeting Gn were comparatively low. Interferon gamma (IFNγ) cellular responses against RVF Gn and Gc glycoproteins were observed in all but one vaccinee in the 5×10^9^ vp dose group. These IFNγ responses peaked at 2 weeks post-vaccination, were highest in the 5×10^10^ vp dose group and tended to be more frequent against the Gn glycoprotein.

**Interpretation:**

ChAdOx1 RVF vaccine was safe, well-tolerated and immunogenic when administered as a single dose in this study population. The data support further clinical development of ChAdOx1 RVF for human use.

**Funding:**

UK Department of Health and Social Care through the UK Vaccines Network; Oak Foundation; Wellcome Trust

## Introduction

Rift Valley fever (RVF) is a mosquito-borne viral zoonosis that primarily affects domestic livestock (sheep, goats, cattle) and humans in Africa and the Arabian Peninsula.^1^ The disease was first identified in Kenya in 1930 and is characterised by high rates (>90%) of death in young animals and abortion (typically >90%) in those that are pregnant.^2^ Spillover into human populations has primarily been attributed to direct contact with infected animal tissues, although mosquito transmission does occur.^2^ Human disease can vary widely; while the majority will experience a self-limiting febrile illness, an estimated 0·5-3% develop severe symptoms, such as haemorrhagic diatheses where case fatality can be as high as 50%.^3^ Other severe complications of RVF such as meningoencephalitis and ocular pathology can lead to debilitating sequelae (e.g. blindness),^3^ while infection during pregnancy carries an increased risk of miscarriage.^4^ Licensed vaccines are available for livestock,^5^ but no licensed RVF vaccines are available for human use. Both the World Health Organization (WHO) and African Union have prioritised RVF for urgent development of vaccines and other countermeasures.^6^

Natural exposure to RVF generates long-lived protective neutralising antibody (nAb) in both humans and livestock, with a modest declining of titres over many years in the absence of re-exposure.^7,8^ When passively transferred into mice, human sera containing nAb confer protection against RVF viral challenge in a dose-dependent manner supporting the importance of nAb in protection.^9^ These nAb target the RVF viral envelope glycoproteins, Gn and Gc, that are well conserved across RVF virus strains and hence provide cross-protective immunity against virus lineages from distant geographic settings.^10^ The goal of RVF vaccinology has therefore been to design vaccines that are safe and highly immunogenic for protective nAb, in addition to meeting other optimal product characteristics defined by the WHO such as the ability to be administered in a single dose, maintenance of immunity over several years, and long-term product stability.^6^ The main target population for a human RVF vaccine are persons resident in areas prone to RVF outbreaks, especially those in contact with livestock including herders, farmers, abattoir workers and veterinarians.^6^ Typically, RVF outbreaks in livestock tend to precede outbreaks in humans underscoring the importance of livestock vaccination to not only minimise animal losses, but also limit virus transmission to humans.^11^ However, this does not obviate the need for a human vaccine because: i) routine livestock vaccination will rarely approach 100% compliance, ii) human cases have been reported in the absence of livestock outbreaks, and iii) there is a potential for epidemic spread.^12^

Two RVF vaccine candidates have previously been evaluated in humans. The first is an inactivated vaccine, TSI-GSD-200, which had a good safety profile but even after an initial three-dose regime, approximately 10% of vaccinees failed to seroconvert.^13^ The second is a live-attenuated vaccine, RVF MP-12. This was investigated in small clinical trials showing a favourable safety and immunogenicity profile in humans,^14^ but further updates on its development have mainly been for its use as a veterinary vaccine.^15^ Over the last few years we have taken a one health approach to RVF vaccinology by developing a single vaccine, ChAdOx1 RVF, for use in both humans and livestock. The vaccine, composed of the ChAdOx1 adenovirus vector expressing a codon-optimised transgene for the RVF viral Gn and Gc glycoproteins (GenBank accession number DQ380208),^16^ utilises the same ChAdOx1 adenovirus vector platform used to make the ChAdOx1 nCoV-19 COVID-19 vaccine that has been deployed in over 180 countries globally,^17^ including Africa where the current burden of RVF disease lies.^1^

ChAdOx1 RVF has shown remarkable safety, immunogenicity and 100% efficacy against wild-type RVF virus challenge in sheep, goats, and cattle in Kenya.^16^ More recently it was found to safely provide protection against disease and foetal loss in pregnant sheep and goats,^18^ supporting its further development for veterinary use. Here, we evaluated the safety, tolerability, and immunogenicity of ChAdOx1 RVF vaccine in a first-in-human phase I trial among healthy adults in the United Kingdom (UK).

## Methods

### Study design and participants

We conducted a phase I, first-in-human, dose-escalation, open-label, non-randomised clinical trial of ChAdOx1 RVF vaccine at the Centre for Clinical Vaccinology and Tropical Medicine, Oxford, UK. Healthy adult volunteers aged 18-55 years were recruited from the local Oxfordshire area using ethically approved advertising materials. Potential volunteers initially completed an online questionnaire covering major exclusion criteria. They were then invited for an in-person screening visit where written informed consent for the study was obtained followed by a medical history assessment, physical examination, urinalysis, and clinical blood tests. Medical histories were corroborated using medical records obtained from the general practitioner (GP) of each volunteer prior to enrolment. Volunteers that had previously been vaccinated with a ChAdOx1 vaccine (e.g. ChAdOx1 nCoV-19) were excluded from the study. Additionally, volunteers with a history of travel to countries endemic for RVF were screened with a commercial RVF ELISA (ID Screen® Multispecies RVFV ELISA, ID.vet) as per manufacturer’s instructions and excluded if seropositive. Results of all screening assessments were reviewed by a trial investigator before enrolment or exclusion. Full eligibility criteria for the trial are detailed in the trial protocol (Supplementary Appendix).

The trial was conducted in accordance with the principles of the Declaration of Helsinki and Good Clinical Practice. Regulatory approval was granted by the UK Medicines and Healthcare Products Regulatory Agency (CTA 21584/0438/001-0001) and ethics approval by National Health Service (NHS) East of England – Cambridge East Research Ethics Committee (reference: 20/EE/0262). Use of ChAdOx1 RVF for this clinical trial was authorised by the Oxford University Hospital NHS Trust Genetic Modification Safety Committee (GM462.18.103). The trial was registered, prior to recruitment, at clinicaltrials.gov (NCT04754776).

### Procedures

The ChAdOx1 RVF vaccine (formerly known as ChAdOx1 GnGc) has been described previously and was manufactured by Advent Srl in accordance with current Good Manufacturing Practices as described in the Investigational Medicinal Product Dossier.^16^ All participants received a single dose of ChAdOx1 RVF, administered intramuscularly into the deltoid of their non-dominant arm. Participants were sequentially (non-randomly) allocated to one of three escalating dose groups, starting with an initial low dose group (5 × 10^9^ virus particles (vp) of ChAdOx1 RVF, n=3), before progression to an intermediate dose group (2·5 × 10^10^ vp, n=6) and finally a high dose group (5 × 10^10^ vp, n=6). A local safety monitor was appointed to provide safety oversight for the trial, who performed the interim safety reviews prior to each dose escalation, containing a minimum of 7 days safety data from all participants of the preceding group. Enrolment was also staggered within groups, with the first participant in each group being vaccinated alone followed by a review at 48 hours prior to enrolment of the next two participants. A further minimum interval of 48 hours was observed before the vaccination of the final three participants in the medium and high dose groups.

Following vaccination, participants attended a series of follow-up visits at the following nominal timepoints: day 2, 7, 14, 28, 56 and 84. Participants also completed a daily online symptom diary for 28 days following vaccination. Solicited adverse events were collected for 7 days and unsolicited adverse events (all other events not defined as solicited) for 28 days post-vaccination. Occurrence of serious adverse events was assessed at all follow up visits. Clinical laboratory blood tests including full blood count, liver function, renal function and electrolytes were performed at day 0 (immediately prior to vaccination), day 2, day 7 and day 28. Laboratory adverse events were graded by use of toxicity tables, which were adapted from the United States Food and Drug Administration toxicity grading scale. Unsolicited adverse events were coded using the Medical Dictionary for Regulatory Activities (MedDRA) version 24·0 and assessed by investigators for causality with ChAdOx1 RVF. Blood samples for immunology assays were taken on day 0 and at days 7, 14, 28, 56 and 84. The schedule of timepoints for all immunogenicity measures was specified in a laboratory analysis plan prior to the enrolment of the first participant. Four timepoint related protocol deviations occurred relating to attendance of visits outside of the planned schedule including 3 participants attending the day 28 visit between 6 and 11 days later and 1 participant attending the day 56 timepoint 17 days earlier than scheduled.

Electronic data capture and clinical data management were carried out using OpenClinica open-source software, version 4·0.

### Outcomes

The primary objective of the study was assessment of safety and tolerability of the vaccine in a healthy adult population. Primary outcome measures were: occurrence of local and systemic solicited adverse events for 7 days after vaccination, occurrence of unsolicited adverse events for 28 days after vaccination, changes in clinical laboratory measures from baseline to day 28, and occurrence of serious adverse events (SAEs) throughout the trial period. The secondary objective was humoral and cellular immunogenicity of the vaccine. Secondary outcome measures were: RVF nAb titres measured against live RVF virus, IgG binding antibody titres measured by enzyme-linked immunosorbent assay (ELISA) against recombinant Gn and Gc proteins, and cellular responses to overlapping peptides spanning the Gn-Gc polyprotein measured by ex-vivo interferon-γ (IFNγ) enzyme-linked immunospot (ELISpot) assay. Exploratory outcomes measures were: IgG1-4 subclass antibody ELISA titres against Gn and Gc proteins, and analysis of correlations between immune parameters. Full details of the immunological assay procedures are in the Supplementary Appendix.

### Statistical analysis

This phase I first-in-human trial aimed to describe the safety, tolerability, and immunogenicity of ChAdOx1 RVF. The number of participants in each vaccine dose group allowed a descriptive analysis of the frequency and magnitude of adverse events following vaccination, rather than statistical significance testing for safety differences between individuals. Immunological data were visualised and analysed using non-parametric tests on GraphPad Prism version 9 (GraphPad Software Inc., California, USA), with a two-sided alpha of 0·05 for statistical significance.

### Role of the funding source

The authors designed, executed, analysed, and reported the study. The funders had no role in these activities other than review of the proposed study design during the funding application.

## Results

**Figure 1:**
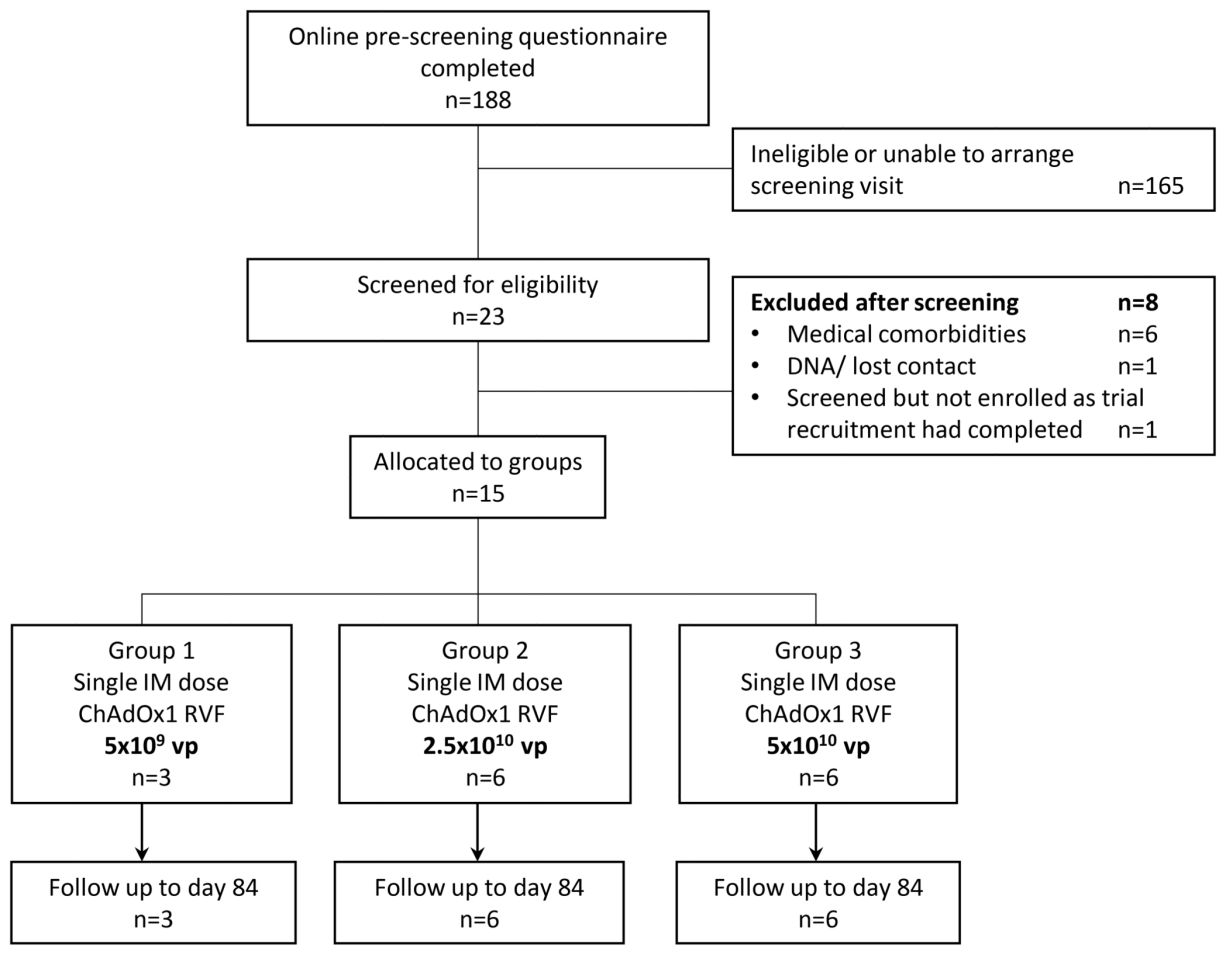
Trial Profile

Between 2^nd^ June 2021 and 7^th^ January 2022, 188 potential volunteers completed our online pre-screening questionnaire, most of whom (n=165) were ineligible or unable to arrange a screening visit. The remaining 23 volunteers were screened for eligibility, of whom 15 eligible volunteers with a median age of 25 years (range 20-38) were enrolled into the study between 11^th^ June 2021 and 13^th^ January 2022 (Figure 1 and Table 1). Nine of the fifteen volunteers were female (Table 1). Three participants were allocated to the low dose group (5 × 10^9^ vp), 6 to the intermediate dose group (2·5 × 10^10^ vp) and 6 to the high dose group (5 × 10^10^ vp; Figure 1). All participants received a single dose of ChAdOx1 RVF vaccine according to their allocated group and were followed up until the final timepoint at day 84. The final follow up visits occurred on 6^th^ April 2022. No major protocol deviations occurred.

ChAdOx1 RVF was determined to have an acceptable safety and tolerability profile during interim safety reviews, allowing dose-escalation to proceed as planned. No serious adverse events occurring in any of the participants following vaccination. Mild local reactions were common, with 14 of the 15 participants reporting solicited local adverse reactions (Table 2). A single participant vaccinated with the intermediate ChAdOx1 RVF dose reported moderate local symptoms (redness, injection site pain and warmth) occurring from day 2 to day 5 post-vaccination but all other participants reported only mild local symptoms. Injection site pain was the most frequently occurring local adverse reaction, occurring in 13 of the 15 participants (Table 2). Local reactions primarily occurred in the early post-vaccination period, with a median onset time of day 1 post vaccination (IQR 0 – 1 days) and median duration of 2 days (IQR 1 – 5 days) (Supplementary Table 1).

No participants in the low dose group reported any systemic solicited adverse events. However, 4 of the 6 participants in the intermediate dose group and all participants in the high dose group reported systemic symptoms that were mostly mild in intensity (Table 2). Systemic reactions were transient and self-limiting, with a median onset time of day 1 post-vaccination (IQR 0 – 1 days) and median duration of 1 day (IQR 1 – 3 days). The most common systemic reactions were headache and fatigue (Table 2). Post-vaccination fever (defined as a temperature greater or equal to 38 °C), self-measured using a provided home thermometer, occurred transiently in 2 high dose participants (who also reported subjective feelings of feverishness) but not in other groups (Table 2). An additional 2 participants in the intermediate dose group reported either mild or moderate subjective feelings of feverishness although with normal measured temperatures (Table 2).

All unsolicited adverse events reported within 28 days of vaccination were either mild or moderate in severity and are detailed within (Supplementary Table 2). Gastrointestinal symptoms were the most common unsolicited adverse reactions (assessed as at least possibly related to vaccination), occurring in 4 of the 15 participants, and were mostly mild in severity although 1 medium dose group participant reported moderate severity diarrhoea and vomiting on day 1 post-vaccination which resolved within 24 hours. Additional moderate severity related adverse reactions were lower limb muscle cramps occurring at day 1 post vaccination only (n=1 medium dose) and worsening of pre-existing dysmenorrhoea symptoms (n=1). Other related adverse reactions included local reactions such as mild lymphadenopathy (n=1 medium dose), vaccine site discomfort (n=1 medium dose), vaccine site joint discomfort (n=1 medium dose) as well as a report of moderate axillary pain (n=1 medium dose).

Mild COVID-19 occurred in 2 participants within 28 days of vaccination, both in the high dose group. The first of these tested positive on a COVID-19 lateral flow device following mild feverishness on day 1 post-vaccination. Their symptoms resolved by day 2 and they were otherwise well. The second volunteer tested positive for COVID-19 after experiencing mild to moderate upper respiratory tract symptoms (sore throat, sneezing, cough) at day 20 which resolved by day 27. Neither of their immune responses to RVFV Gn and Gc was remarkable in comparison to other participants and it is unclear how, if at all, their COVID-19 infections impacted upon this.

Laboratory adverse events are described in Supplementary Table 3. Transient decreases in total white cell, lymphocyte or neutrophil counts occurred at day 2 in some of the intermediate and high dose group participants. Lymphopenia graded as severe occurred in 2 high dose participants, both occurring at a single timepoint only: day 2 or 7. These fully resolved at the subsequent follow up visit timepoint in both cases. No platelet count abnormalities were seen in any participants during the study. Hypokalaemia graded as severe was recorded at a single timepoint (day 28) in a single volunteer who had otherwise normal clinical biochemical markers and was well. This was assessed as not causally related to vaccination and attributed to pseudohypokalaemia by investigators due to the use of lithium heparin blood tubes and a 12-hour delay from venepuncture to clinical blood sample processing occurring in this instance.

**Figure 2:**
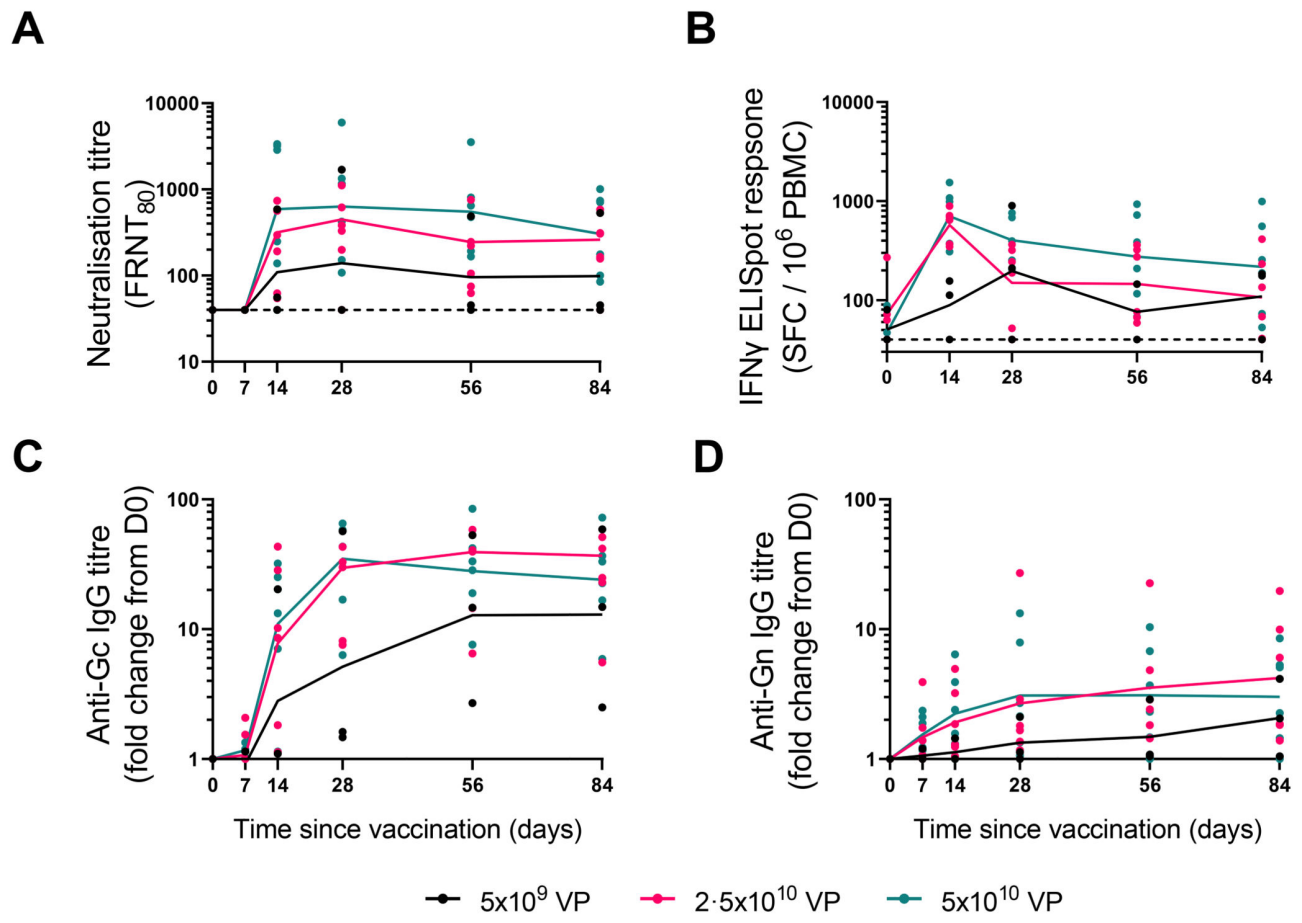
Humoral and cellular responses generated by ChAdOx1 RVF vaccination ChAdOx1 RVF vaccine immunogenicity kinetics are shown for all participants (n=15) by dose allocation and at all immunology sampling timepoints during the 3 months of follow up. RVF nAb titres are shown in (A), summed Gn and Gc IFN γ ELISpot responses in (B), and total IgG response against Gc (C) and Gn (D) are shown as fold change from baseline (D0). Symbols represent mean values from three replicates, while connecting lines represent median values. Four samples were obtained out of the defined timepoint windows: 2.5x10^10^ vp group (n=1 D28 sample taken 10 days after the timepoint, n=1 D56 sample taken 17 days before the timepoint), 5x10^10^ vp group (n=1 D28 sample taken 6 days after the timepoint, n=1 D28 sample taken 11 days after the timepoint).

ChAdOx1 RVF was highly immunogenic, with 12 of the 15 vaccinees mounting a detectable RVF nAb response that peaked at day 28 post-vaccination and persisted to the final follow-up visit at day 84 (Figure 2). The 3 volunteers that failed to mount a nAb response were either in the low dose (n=2) or intermediate dose group (n=1), respectively (Figure 2). Binding IgG titres targeting Gc tended to peak at day 28, remaining high over the 3-month follow-up period (Figure 2). This was predominantly driven by strong IgG1 and IgG3 responses, with no significant induction of IgG2 and IgG4 detected (Supplementary Figure 1). There was a strong correlation between titres of binding IgG targeting Gc and their ability to neutralise RVF virus (Spearman correlation; r = 0·84 [95%CI 0.55-0.95], (Supplementary Figure 2). Binding IgG titres towards Gn were comparatively poor, with a very modest increase in the median response after vaccination in all dose groups (Figure 2). This was underlined by minimal detection of any increase in the IgG subclasses post-vaccination and poor correlation with nAb titres (Spearman correlation; r = 0·39 [-0.17-0.76]) (Supplementary Figure 1). Two volunteers from the low dose (n=1) and high dose groups (n = 1) had significant non-specific IgG binding Gn, with prevaccination responses being higher than the median post-vaccination peak response; neither increased their response post-vaccination. These volunteers showed no neutralising activity at day 0, a highly specific assay, suggesting significant cross-reactivity explains the high Gn ELISA background. ChAdOx1 RVF elicited a dose-dependent IFNγ ELISpot response in 14 of the 15 vaccinees (Figure 2). Only a single volunteer in the low dose group failed to mount any detectable IFNγ response. Responses in the low dose group peaked at day 28 post-vaccination, with a median of 212 spot-forming cells (SFC)/10^6^ PBMC (IQR 126-556). All intermediate and high dose vaccinees mounted a response that peaked at day 14 post-vaccination with median responses of 655 (IQR 437-706) and 810 (IQR 441-1054) SFC/10^6^ PBMC, respectively (Figure 2). IFNγ responses were broad, with all Gn and Gc peptide pools stimulating an IFNγ response in at least one individual (Supplementary Figure 1). Overall, peak IFNγ responses were higher in magnitude towards Gn than they were towards Gc (Supplementary Figure 1). There was no statistically significant correlation between IFNγ ELISpot response and nAb titre (Spearman correlation; r = 0·29 [-0.30-0.72]) (Supplementary Figure 2). Of the three volunteers that failed to develop nAb activity, one (from the low dose group) showed no IFNγ response either while two of them (one from the low dose group and the other from the intermediate dose group) had comparatively strong IFNγ responses (Figure 2).

## Discussion

There are currently no vaccines for use against RVF in humans, leaving the world vulnerable to public health emergencies associated with RVF epidemics. For this reason, the WHO has prioritised development of RVF countermeasures and compiled a target product profile to guide vaccine development.^6^ In this study we show that ChAdOx1 RVF meets many of the optimal product characteristics listed in the WHO target product profile for a human RVF vaccine.^6^ These include a favourable safety profile with predominantly mild or transient adverse effects and a rapid onset of RVF nAb and cellular immunity within 2 weeks of single-dose vaccination. Adverse events observed in this first-in-human trial were consistent with the known safety profile of ChAdOx1 and other adenoviral vectored vaccines. These primarily consisted of mild local or systemic reactions generally starting and resolving within 48 hours of administration of the vaccine. The highly transient but marked lymphopenia observed in two participants at the high dose has frequently been reported with other vaccines, appears to be a benign phenomenon, and has been suggested to be caused by the redistribution of lymphocytes into lymphoid tissue.^19^ These safety and immunological attributes of ChAdOx1 RVF following single dose vaccination are critical for reactive vaccination during RVF outbreaks.^6^ Its inherent thermostability, allowing storage at fridge temperatures for at least a year without loss of potency, should allow relatively straightforward deployment.^20^

A scalable manufacturing process for ChAdOx1-vectored vaccines has been developed and successfully used for the ChAdOx1 nCoV-19 (AZD1222) COVID-19 vaccine that is now deployed in over 180 countries globally,^17^ and this should be readily applicable for ChAdOx1 RVF. With a large proportion of the world’s population having received ChAdOx1 nCoV-19, including in areas where RVF vaccines are likely to be useful, there is a legitimate concern that multiple doses of homologous viral vectors could be problematic due to the build-up of anti-vector immunity. However, evidence from previous ChAdOx1 vaccine trials suggests that neither prior doses of ChAdOx1 vaccines nor naturally acquired anti-ChAdOx1-vector neutralising antibodies lead to impaired immune responses to the encoded antigen.^21^

Post-marketing surveillance of adenovirus-vectored COVID-19 vaccines, including ChAdOx1 nCoV-19, uncovered a very rare association with thrombosis with thrombocytopenia syndrome (TTS). The biological mechanism underlying TTS is incompletely understood and it remains unknown whether it may also affect adenovirus-vectored vaccines that do not deliver coronavirus antigens.^22^ Very few cases of TTS have been reported outside of North America, Europe and Australia despite widespread use of adenovirus-vectored COVID-19 vaccines around the world. This regional disparity in TTS incidence may in part represent underreporting due to difficulties in case identification and pharmacosurveillance, but in some countries with excellent pharmacovigilance TTS appears very rare.^22^

In keeping with our previous livestock studies,^16,18^ a single dose of ChAdOx1 RVF elicited high RVF nAb titres in humans with the highest titres being observed in the high dose group (5 x 10^10^ vp), which is likely to be the preferred dose for later phase studies. The nAb response persisted to the end of follow up at 3 months in all individuals that seroconverted post-vaccination, irrespective of vaccine dose. While the precise nAb titre that correlates with protection against RVF is yet to be established, it appears to be very low, with very few instances in the literature where an animal or human carrying RVF nAb of any level has developed clinical RVF.^16,23–25^ Studies attempting to investigate the minimal protective nAb titre using rodent models have confirmed this. An early study in hamsters after vaccination or adoptive transfer with human immune serum found nAb titres (as measure by plaque reduction neutralisation test [PRNT]) of between 10-20 offered full protection^26^. A more recent study using adoptive transfer of serum from human MP-12 vaccine recipients into mice,^27^ showed PRNT titres as low as 1:5 appear largely protective. Our own data from livestock vaccinated with ChAdOx1 RVF has shown complete protection from challenge, even in animals with titres that are orders of magnitude lower than the highest responders.^16^ Unfortunately, comparisons of nAb titres elicited after different RVF vaccine candidates are difficult without harmonised assays or a shared serum standard. We are, however, among a number of laboratories participating in the establishment of the first WHO International Standard for anti-RVFV antibody, which will facilitate comparisons with different vaccine candidates in the future. These data underlie the use of viral envelope glycoproteins Gn and Gc (both targets of nAb)^8,28^ as the main components of candidate RVF vaccines in development.^5,6,10^ These envelope glycoproteins are important for viral attachment and entry into cells and exhibit limited genetic diversity such that nAb generated by vaccination or natural infection provide cross-protection against heterologous virus strains/lineages.^8,29^

The short follow-up duration of this first-in-human trial precluded durability assessments of immune responses generated by vaccination. However, the high nAb titres and the robust IFNγ cellular response detected within 2 weeks post-vaccination augur well for reactive use of the vaccine during RVF outbreaks where rapid induction of immunity is necessary to protect individuals at highest risk of exposure.^6^ Future phase 2 trial with long-term follow-up in populations resident in RVF epidemic-prone settings will determine the durability of the vaccine-induced nAb response, its relationship with T cell responses and whether homologous prime-boost regimens would provide any benefit in the magnitude of the memory B cell frequencies as compared to the single-dose regime. The relevance of the observed predominance of anti-Gc humoral response to vaccination, despite a strong IFNγ response targeting both Gn and Gc peptides, will need further investigation in future studies. Whether a particular nAb titre is made up primarily of antibodies targeting Gc rather than Gn or vice versa is unlikely to be of significant consequence with regards to efficacy. The discrepancy seen here could be an indication that, while Gn is being expressed as evidenced by the IFNγ ELISpot response, perhaps its tertiary structure differs from the native protein as it appears on the virion surface. If so, then there is potential to significantly increase the nAb response if this could be rectified. On the other hand, if Gc expressed from this vaccine is significantly more accessible than its counterpart on the native virion surface, it may be inevitable that we induce antibodies predominantly towards Gc. Comparisons between immunity induced by vaccination and immunity acquired from natural RVF infection will help define key mechanisms that underlie protection,^8^ and determine whether hybrid immunity has an impact on immunological attributes of the memory B cell response as recently observed for SARS-CoV-2.^30^

This first-in-human trial had several limitations, including the small sample size which was sufficient for informing decisions on further evaluation in phase 2 trial but not for detection of any rare adverse events. Subsequent trials will allow assessment of any rare adverse events associated with vaccination. Long-term durability of the immune response could not be determined due to the short follow-up duration. In addition, the study participants were predominantly of white ethnicity, in a population where RVF is not endemic. The four visits conducted outside of the planned trial visit schedule may also have added variability to the measured immune responses. To generalise vaccine performance to populations at most risk, ChAdOx1 RVF and other candidate human RVF vaccines will need evaluation in the target populations in RVF-endemic regions and including adults, children and adolescents, who are all involved in animal husbandry, and in pregnant women where risk of RVF-associated miscarriage is high.^4^

## Supplementary Material

Supplementary materials

## Figures and Tables

**Table 1:** Baseline Characteristics

	5 x 10^9^ vp(n=3)	2 x 5 × 10^10^vp (n=6)	5 x 10^10^ vp (n=6)	All groups (n=15)
**Sex**				
Female	1	4	4	9
Male	2	2	2	6
**Age in years** (median, range)	25 (20-29)	27 (21-31)	24.5 (20-38)	25 (20-38)
**Ethnicity**				
White	2	5	5	12
Asian	-	-	1	1
Black	-	1	-	1
Mixed White and Black Caribbean	1	-	-	1

**Table 2:** Solicited adverse events within 7 days of vaccination with ChAdOx1 RVF

	5 x 10^9^ vp (n=3)			2·5 x 10^10^vp (n=6)			5 x 10^10^ vp (n=6)		
	Mild	Moderate	Severe	Mild	Moderate	Severe	Mild	Moderate	Severe
**Any Symptom**	2 (67%)	0	0	4 (67%)	2 (33%)	0	5 (83%)	1 (17%)	0
**Any Local Symptom**	2 (67%)	0	0	5 (83%)	1 (17%)	0	6 (100%)	0	0
Pain	2 (67%)	0	0	4 (67%)	1 (17%)	0	6 (100%)	0	0
Redness	0	0	0	1 (17%)	1 (17%)	0	1 (17%)	0	0
Warmth	2 (67%)	0	0	4 (67%)	1 (17%)	0	1 (17%)	0	0
Itch	1 (33%)	0	0	2 (33%)	0	0	0	0	0
**Any Systemic Symptom**	1 (33%)	0	0	2 (33%)	2 (33%)	0	5 (83%)	1 (17%)	0
Fever	0	0	0	0	0	0	1 (17%)	1 (17%)	0
Feverishness	0	0	0	1 (17%)	1 (17%)	0	1 (17%)	1 (17%)	0
Arthralgia	0	0	0	3 (50%)	0	0	2 (33%)	0	0
Myalgia	0	0	0	2 (33%)	0	0	2 (33%)	0	0
Fatigue	0	0	0	3 (50%)	1 (17%)	0	2 (33%)	1 (17%)	0
Headache	0	0	0	3 (50%)	1 (17%)	0	4 (67%)	1 (17%)	0
Nausea	0	0	0	2 (33%)	1 (17%)	0	0	0	0
Malaise	0	0	0	2 (33%)	2 (33%)	0	1 (17%)	1 (17%)	0

## Data Availability

Deidentified participant data will be made available upon requests directed to the chief investigator. Proposals will be reviewed and approved by the sponsor, chief investigator, and collaborators on the basis of scientific merit. After approval of a proposal, data can be shared through a secure online platform after signing a data access agreement. The trial protocol is included in the supplementary materials.

## Research in context

### Evidence before this study

Both the WHO and African Union have identified vaccine development for RVF in humans as an urgent priority owing to its potential to cause a public health emergency with devastating health consequences and major economic impacts. The goal of RVF vaccinology has been to design vaccines that are safe and highly immunogenic for neutralizing antibody (nAb) - which are associated with protection - in addition to other optimal product characteristics defined by the WHO. We conducted a search for RVF vaccine trials on ClinicalTrials.gov (using: (Rift Valley Fever) AND (vaccine) with no date restrictions). Two candidate vaccines, the inactivated TSI-GSD-200 vaccine, and the live-attenuated MP-12 vaccine, have previously been evaluated in humans and shown to safely elicit nAb. TSI-GSD-200 has been registered on ClinicalTrials.gov as currently recruiting for phase 2 trial but there have been no further updates on the clinical development of MP-12 for human use since completion of a phase 2 trial in 2009. ChAdOx1 RVF, which is described in this manuscript is also listed as undergoing a separate phase 1b trial in Uganda (registration number:NCT04672824). As with TSI-GSD-200 and MP-12, further evaluation of ChAdOx1 RVF in humans follows demonstration of safety, immunogenicity and efficacy against RVF in livestock.

### Added value of this study

This study describes a first-in-human trial of ChAdOx1 RVF, a chimpanzee adenovirus vectored RVF vaccine that has been shown to be highly immunogenic and efficacious against RVF in all major livestock species affected by the disease. While the ChAdOx1 platform is widely deployed for use against COVID-19 (Oxford/AstraZeneca AZD1222 vaccine) in over 180 countries globally, this is the first clinical evaluation of its use for RVF in humans. We assessed the safety and immunogenicity of a single intramuscular dose of ChAdOx1 RVF among adults in the UK. ChAdOx1 RVF was well tolerated with no serious adverse events. High RVF nAb titres were detected within 2 weeks of vaccination, peaking at 28 days post-vaccination. A strong RVF viral glycoprotein-specific IFNγ response, peaking 2 weeks post-vaccination, was also detected. Both humoral and cellular responses persisted over the 3 months follow up period of the study.

### Implications of all the available evidence

A vaccine for use against RVF in humans remains an urgent unmet need. ChAdOx1 has been shown to be a scalable vaccine platform for COVID-19, but this is the first use of the platform for RVF in humans. ChAdOx1 RVF was well tolerated and generated strong humoral and cellular immune responses. Further evaluation of the vaccine in populations at most risk for RVF is warranted.

## References

[1] Bron GM, Strimbu K, Cecilia H (2021). Over 100 Years of Rift Valley Fever: A Patchwork of Data on Pathogen Spread and Spillover. Pathogens.

[2] Daubney R, Hudson JR, Granham PC (1931). Enzootic Hepatitis or Rift Valley fever: an undescribed virus disease of sheep, cattle and man from East Africa. Journal of Pathology and Bacteriology.

[3] Anywaine Z, Lule SA, Hansen C, Warimwe G, Elliott A (2022). Clinical manifestations of Rift Valley fever in humans: Systematic review and meta-analysis. PLoS Negl Trop Dis.

[4] Baudin M, Jumaa AM, Jomma HJE (2016). Association of Rift Valley fever virus infection with miscarriage in Sudanese women: a cross-sectional study. Lancet Glob Health.

[5] Dungu B, Lubisi BA, Ikegami T (2018). Rift Valley fever vaccines: current and future needs. Curr Opin Virol.

[6] WHO (2019). Target Product Profiles for Rift Valley Fever virus vaccines.

[7] Brown RD, Scott GR, Dalling T (1957). Persistence of antibodies to Rift Valley Fever in man. Lancet.

[8] Wright D, Allen ER, Clark MHA (2020). Naturally Acquired Rift Valley Fever Virus Neutralizing Antibodies Predominantly Target the Gn Glycoprotein. iScience.

[9] Doyle JD, Barbeau DJ, Cartwright HN, McElroy AK (2022). Immune correlates of protection following Rift Valley fever virus vaccination. NPJ Vaccines.

[10] Wright D, Kortekaas J, Bowden TA, Warimwe GM (2019). Rift Valley fever: biology and epidemiology. J Gen Virol.

[11] Warimwe GM, Francis MJ, Bowden TA, Thumbi SM, Charleston B (2021). Using cross-species vaccination approaches to counter emerging infectious diseases. Nat Rev Immunol.

[12] Baba M, Masiga DK, Sang R, Villinger J (2016). Has Rift Valley fever virus evolved with increasing severity in human populations in East Africa?. Emerg Microbes Infect.

[13] Pittman PR, Liu CT, Cannon TL (1999). Immunogenicity of an inactivated Rift Valley fever vaccine in humans: a 12-year experience. Vaccine.

[14] Pittman PR, McClain D, Quinn X (2016). Safety and immunogenicity of a mutagenized, live attenuated Rift Valley fever vaccine, MP-12, in a Phase 1 dose escalation and route comparison study in humans. Vaccine.

[15] Nyundo S, Adamson E, Rowland J (2019). Safety and immunogenicity of Rift Valley fever MP-12 and arMP-12DeltaNSm21/384 vaccine candidates in goats (Capra aegagrus hircus) from Tanzania. Onderstepoort J Vet Res.

[16] Warimwe GM, Gesharisha J, Carr BV (2016). Chimpanzee Adenovirus Vaccine Provides Multispecies Protection against Rift Valley Fever. Sci Rep.

[17] Joe CCD, Jiang J, Linke T (2022). Manufacturing a chimpanzee adenovirus-vectored SARS-CoV-2 vaccine to meet global needs. Biotechnol Bioeng.

[18] Stedman A, Wright D, Wichgers Schreur PJ (2019). Safety and efficacy of ChAdOx1 RVF vaccine against Rift Valley fever in pregnant sheep and goats. NPJ Vaccines.

[19] Sahin U, Muik A, Derhovanessian E (2020). COVID-19 vaccine BNT162b1 elicits human antibody and TH1 T cell responses. Nature.

[20] Berg A, Wright D, Dulal P (2021). Stability of Chimpanzee Adenovirus Vectored Vaccines (ChAdOx1 and ChAdOx2) in Liquid and Lyophilised Formulations. Vaccines (Basel).

[21] Emary KRW, Golubchik T, Aley PK (2021). Efficacy of ChAdOx1 nCoV-19 (AZD1222) vaccine against SARS-CoV-2 variant of concern 202012/01 (B.1.1.7): an exploratory analysis of a randomised controlled trial. Lancet.

[22] Buoninfante A, Andeweg A, Baker AT (2022). Understanding thrombosis with thrombocytopenia syndrome after COVID-19 vaccination. NPJ Vaccines.

[23] Kortekaas J, Antonis AF, Kant J (2012). Efficacy of three candidate Rift Valley fever vaccines in sheep. Vaccine.

[24] Faburay B, Wilson WC, Gaudreault NN (2016). A Recombinant Rift Valley Fever Virus Glycoprotein Subunit Vaccine Confers Full Protection against Rift Valley Fever Challenge in Sheep. Sci Rep.

[25] Bird BH, Maartens LH, Campbell S (2011). Rift Valley fever virus vaccine lacking the NSs and NSm genes is safe, nonteratogenic, and confers protection from viremia, pyrexia, and abortion following challenge in adult and pregnant sheep. J Virol.

[26] NIKLASSON BS, MEADORS GF, PETERS CJ (1984). Active and passive immunization against Rift Valley fever virus infection in Syrian hamsters. Acta Pathologica Microbiologica Scandinavica Series C: Immunology.

[27] Watts DM, Westover JL, Palermo PM (2022). Estimation of the Minimal Rift Valley Fever Virus Protective Neutralizing Antibody Titer in Human Volunteers Immunized with MP-12 Vaccine Based on Protection in a Mouse Model of Disease. The American Journal of Tropical Medicine and Hygiene.

[28] Besselaar TG, Blackburn NK (1991). Topological mapping of antigenic sites on the Rift Valley fever virus envelope glycoproteins using monoclonal antibodies. Arch Virol.

[29] Besselaar TG, Blackburn NK, Meenehan GM (1991). Antigenic analysis of Rift Valley fever virus isolates: monoclonal antibodies distinguish between wild-type and neurotropic virus strains. Research in virology.

[30] Crotty S (2021). Hybrid Immunity. Science.

