# Supplementary material for: Safety and immunogenicity of a ChAdOx1 vaccine against Rift Valley Fever in adults: an open-label, non-randomised, first-in-human phase 1 clinical trial
Source: Lancet Infect Dis. Author manuscript; Available in PMC 2023 Aug 1. (PMC7614834; doi:10.1016/S1473-3099(23)00068-3)
Supplement: Supplementary materials [file EMS164638-supplement-Supplementary_materials.pdf]

SUPPLEMENTARY MATERIALS

Table of Contents

Supplementary Laboratory Methods........2
Enzyme-linked immunosorbent assay (ELISA)........2
RVF virus neutralisation assay........2
Enzyme-linked immunospot assay (ELISpot)........2
Supplementary Table 1: Onset time and duration of solicited adverse events........3
Supplementary Table 2: Unsolicited adverse events........4
Supplementary Table 3: Clinical laboratory graded adverse events........6
Supplementary Figure 1: Supplementary Immunology........7
Supplementary Figure 2: Correlations between immune parameters........8
Supplementary Materials References........9
RVF001 Clinical Trial Protocol........10

Supplementary Laboratory Methods

Enzyme-linked immunosorbent assay (ELISA)

Recombinant RVFV Gn ectodomain (UniProt accession number P21401, residues 154–560) and Gc ectodomain (UniProt accession number P21401, residues 691–1120) were expressed in HEK293 cells as previously described [1]. This protein was used to perform ELISAs as previously described [1] with the exception of the following modifications. Plates were blocked with 100uL/well of ChonBlock™. Samples were diluted in ChonBlock™ before addition to plate. The secondary antibody used (goat anti-human whole IgG-alkaline phosphatase, Sigma, St. Louis, MO, USA) was diluted 1:1000 in ChonBlock™. Plates were developed by adding 100 µL/well of 4-nitrophenyl phosphate disodium salt Hexahydrate (Sigma) in diethanolamine buffer (ThermoScientific). Optical density (OD) was read at 405 nm using a BioTek ELx808 plate reader until the internal control (reference serum diluted 1:800) had reached an OD of 1. OD values of reference serum titrations were then fitted to a 4-parameter standard curve using Gen5 software (v3.09 BioTek). Test sera antibody units were calculated from their OD values using the estimated parameters from the standard curve. IgG1-4 subclass responses were assayed in the same way, using the secondary antibodies described here[2]. All samples for a particular IgG subclass were run on the same plate at the same time, with raw OD values used to calculate fold change from D0:D28."

RVF virus neutralisation assay

RVFV neutralisation was assessed using a focus reduction neutralisation test (FRNT) as previously described with some alterations.[1] Briefly, serum was diluted in DMEM containing 10% foetal bovine serum, 1% L-glutamine and 1% penicillin/streptomycin (D10) in 2-fold steps in duplicate from a starting dilution of 1:20, 100 µL/well. Each plate contained a negative control serum and a positive control serum in duplicate. The live attenuated RVFV-4s-eGFP was diluted to 10,000 TCID50 / mL in D10 and 100 µL/well added.[3] Each plate also contained media-only wells in duplicate (200 µL media, no virus) in addition to 10 wells of virus-only (100 µL media + 100 µL virus). Plates were incubated for 1h at 37 °C before transferring serum/virus mixture to 96-well TC plates containing Vero E6 monolayers at >80% confluence (seeded the day before at $2x10^5$ cells/mL in D10, 50 µL/well). TC plates were incubated for 15-18h in 37 °C, 5% CO2. Plates were then developed as described previously.[1] Serum dilution required to reduce virus foci by 80% ($FRNT_{80}$) compared to virus-only control wells (10 replicates per plate) was calculated from a 4-parameter standard curve using GraphPad Prism version 9 (GraphPad Software Inc., California, USA).

Enzyme-linked immunospot assay (ELISpot)

We assessed the IFNγ ELISpot response after stimulation of PBMC samples, freshly isolated from whole blood, with an overlapping peptide library spanning Gn and Gc. 264 15mer peptides overlapping by 11 amino acids, covering the Gn-Gc polyprotein (GenBank accession number DQ380208, residues 132-1197) were synthesised (ProImmune, Oxford, UK). Peptides were split into 10 pools of 25/26 peptides each at 10 µg/mL final concentration - 5 pools covering Gn and 5 pools covering Gc. ELISpots were carried out as previously described.[4] Responses from unstimulated wells were subtracted before quantifying the response to the 10 pools and summing them. Positive control wells were stimulated with anti-CD3 antibody (Cambridge Bioscience Ltd) at 100 ng/mL.

Supplementary Table 1: Onset time and duration of solicited adverse events
Summary of duration in days and post vaccination onset time in days of reported solicited AEs

**AE**	**Duration [Days] median (IQR)**	**Onset [Days post vaccination] median (IQR)**
Redness	1 (1 - 3)	1 (0.5 - 1.5)
Pain	3 (2 - 5)	1 (0 - 1)
Itch	1 (1 - 3.5)	4 (2 - 5)
Warmth	1 (1 - 2.75)	1 (1 - 1)
Fever	1 (1 - 1)	0.5 (0.25 - 0.75)
Arthralgia	1 (1 - 2)	1 (1 - 1)
Myalgia	1 (1 - 1.5)	0.5 (0 - 1)
Feverishness	1 (1 - 1.25)	0.5 (0 - 1)
Headache	1 (1 - 2)	1 (0 - 1)
Fatigue	1 (1 - 3.5)	1 (0 - 1)
Nausea	1 (1 - 3)	1 (1 - 1)
Malaise	2 (1.25 - 5)	0.5 (0 - 1)
Any Local	2 (1 - 5)	1 (0 - 1)
Any Systemic	1 (1 - 2)	1 (0 - 1)
Any AE	1 (1 - 3)	1 (0 - 1)

Supplementary Table 2: Unsolicited adverse events

Number and percentage of participants reporting related or unrelated unsolicited adverse events reported within 28 days of vaccination. Presented by relatedness, MEDDRA System Organ Classes (SOC), MEDDRA Preferred Terms (PT) and severity grade.

Unsolicited AEs within 28 days of ChAdOx1 RVF	5 x 10^9 vp (n=3)	2·5 × 10^{10} vp (n=6)	5 x 10^{10} vp (n=6)	All groups (n=15)
**AEs assessed as "possibly", "probably" or "definitely" related to vaccination**	**1 (33%)**	**4 (67%)**	**2 (33%)**	**7 (47%)**
**Blood and lymphatic system disorders**	**0**	**1 (17%)**	**0**	**1 (7%)**
Lymphadenopathy	0	1 (17%)	0	1 (7%)
Grade 1	0	1 (17%)	0	1 (7%)
**Gastrointestinal disorders**	**1 (33%)**	**2 (33%)**	**1 (17%)**	**4 (27%)**
Abdominal pain	0	0	1 (17%)	1 (7%)
Grade 1	0	0	1 (17%)	1 (7%)
Diarrhoea	1 (33%)	1 (17%)	1 (17%)	3 (20%)
Grade 1	1 (33%)	0	1 (17%)	2 (13%)
Grade 2	0	1 (17%)	0	1 (7%)
Vomiting	0	1 (17%)	0	1 (7%)
Grade 2	0	1 (17%)	0	1 (7%)
**General disorders and administration site conditions**	**0**	**3 (50%)**	**0**	**3 (20%)**
Axillary pain	0	1 (17%)	0	1 (7%)
Grade 2	0	1 (17%)	0	1 (7%)
Chills	0	2 (33%)	0	2 (13%)
Grade 1	0	1 (17%)	0	1 (7%)
Grade 2	0	1 (17%)	0	1 (7%)
Vaccination site discomfort	0	1 (17%)	0	1 (7%)
Grade 1	0	1 (17%)	0	1 (7%)
Vaccination site joint discomfort	0	1 (17%)	0	1 (7%)
Grade 1	0	1 (17%)	0	1 (7%)
**Metabolism and nutrition disorders**	**0**	**1 (17%)**	**0**	**1 (7%)**
Decreased appetite	0	1 (17%)	0	1 (7%)
Grade 1	0	1 (17%)	0	1 (7%)
**Musculoskeletal and connective tissue disorders**	**0**	**1 (17%)**	**0**	**1 (7%)**
Muscle spasms	0	1 (17%)	0	1 (7%)
Grade 2	0	1 (17%)	0	1 (7%)
**Reproductive system and breast disorders**	**0**	**1 (17%)**	**0**	**1 (7%)**
Dysmenorrhoea	0	1 (17%)	0	1 (7%)
Grade 2	0	1 (17%)	0	1 (7%)
**Respiratory, thoracic and mediastinal disorders**	**0**	**0**	**1 (17%)**	**1 (7%)**
Sore throat	0	0	1 (17%)	1 (7%)
Grade 1	0	0	1 (17%)	1 (7%)
**AEs assessed as "no relationship" or "unlikely related" to vaccination**	**0**	**2 (33%)**	**5 (83%)**	**7 (47%)**
**Gastrointestinal disorders**	**0**	**0**	**1 (17%)**	**1 (7%)**
Abdominal discomfort	0	0	1 (17%)	1 (7%)
Grade 1	0	0	1 (17%)	1 (7%)
Dyspepsia	0	0	1 (17%)	1 (7%)
Grade 1	0	0	1 (17%)	1 (7%)
**Infections and infestations**	**0**	**1 (17%)**	**3 (50%)**	**4 (27%)**
COVID-19	0	0	2 (33%)	2 (13%)
Grade 1	0	0	1 (17%)	1 (7%)
Grade 2	0	0	1 (17%)	1 (7%)
Pustule	0	1 (17%)	0	1 (7%)
Grade 2	0	1 (17%)	0	1 (7%)
Rhinitis	0	0	1 (17%)	1 (7%)
Grade 1	0	0	1 (17%)	1 (7%)
Urinary tract infection	0	1 (17%)	0	1 (7%)
Grade 2	0	1 (17%)	0	1 (7%)
**Investigations**	**0**	**1 (17%)**	**0**	**1 (7%)**
Blood pressure increased	0	1 (17%)	0	1 (7%)
Grade 2	0	1 (17%)	0	1 (7%)
**Musculoskeletal and connective tissue disorders**	**0**	**1 (17%)**	**0**	**1 (7%)**
Axillary mass	0	1 (17%)	0	1 (7%)
Grade 2	0	1 (17%)	0	1 (7%)
**Nervous system disorders**	**0**	**2 (33%)**	**2 (33%)**	**4 (27%)**
Headache	0	1 (17%)	2 (33%)	3 (20%)
Grade 1	0	1 (17%)	2 (33%)	3 (20%)
Hypoaesthesia	0	1 (17%)	0	1 (7%)

Grade 1	0	1 (17%)	0	1 (7%)
**Respiratory, thoracic and mediastinal disorders**	**0**	**1 (17%)**	**1 (17%)**	**2 (13%)**
Dyspnoea	0	1 (17%)	0	1 (7%)
Grade 2	0	1 (17%)	0	1 (7%)
Epistaxis	0	0	1 (17%)	1 (7%)
Grade 1	0	0	1 (17%)	1 (7%)
**Total Unsolicited AEs**	**1 (33%)**	**4 (67%)**	**6 (100%)**	**11 (73%)**

Supplementary Table 3: Clinical laboratory graded adverse events

Clinical laboratory results obtained measured at each evaluated timepoint in the trial for all groups. Abnormal results were graded according to a pre-specified laboratory adverse events severity grading scale.

**Group**	**5 x 10^9 vp (n=3)**				**2·5 × 10^{10} vp (n=6)**					**5 x 10^{10} vp (n=6)**			
**Timepoint (day)**	**0**	**2**	**7**	**28**	**0**	**2**	**7**	**28**	**Extra Visit**	**0**	**2**	**7**	**28**
**Number evaluated (n)**	**3**	**3**	**3**	**3**	**6**	**6**	**6**	**5**	**1**	**6**	**5**	**6**	**6**
**Albumin**													
normal	3	3	3	3	6	6	6	5	1	6	5	6	6
**Alk. Phos.**													
normal	3	3	3	3	6	6	6	5	1	6	5	6	6
**ALT**													
normal	3	3	3	3	6	6	6	5	1	5	4	6	6
Grade 1 raised ALT	-	-	-	-	-	-	-	-	-	1	1	-	-
**Bilirubin**													
normal	3	2	2	2	5	6	6	4	1	6	5	6	6
Grade 1 raised bilirubin	-	-	1	1	1	-	-	1	-	-	-	-	-
Grade 2 raised bilirubin	-	1	-	-	-	-	-	-	-	-	-	-	-
**Creatinine**													
normal	3	3	3	3	6	6	6	5	1	6	5	6	6
**Potassium**													
normal	3	1	2	3	6	6	6	4	1	5	5	6	6
Grade 1 raised potassium	-	2	1	-	-	-	-	-	-	1	-	-	-
Grade 3 raised potassium	-	-	-	-	-	-	-	1	-	-	-	-	-
**Sodium**													
normal	3	3	3	3	6	6	6	5	1	6	5	6	6
**Urea**													
normal	3	3	3	3	6	6	6	5	1	6	5	6	6
**Eosinophil**													
normal	3	3	3	3	6	6	6	5	1	6	5	6	6
**Haemoglobin**													
normal	3	3	3	3	6	6	6	5	1	6	5	6	6
**Lymphocyte**													
normal	3	3	3	3	6	5	6	5	-	5	3	5	6
Grade 2 decreased lymphocytes	-	-	-	-	-	1	-	-	1	1	1	-	-
Grade 3 decreased lymphocytes	-	-	-	-	-	-	-	-	-	-	1	1	-
**Neutrophils**													
normal	3	3	3	3	5	4	5	4	1	6	4	6	6
Grade 1 decreased neutrophils	-	-	-	-	1	1	1	1	-	-	1	-	-
Grade 2 decreased neutrophils	-	-	-	-	-	1	-	-	-	-	-	-	-
**Platelets**													
normal	3	3	3	3	6	6	6	5	1	6	5	6	6
**White Cell Count**													
normal	3	3	3	3	6	3	6	4	1	6	4	6	6
Grade 1 decreased WBC	-	-	-	-	-	3	-	1	-	-	1	-	-

Supplementary Figure 1: Supplementary Immunology

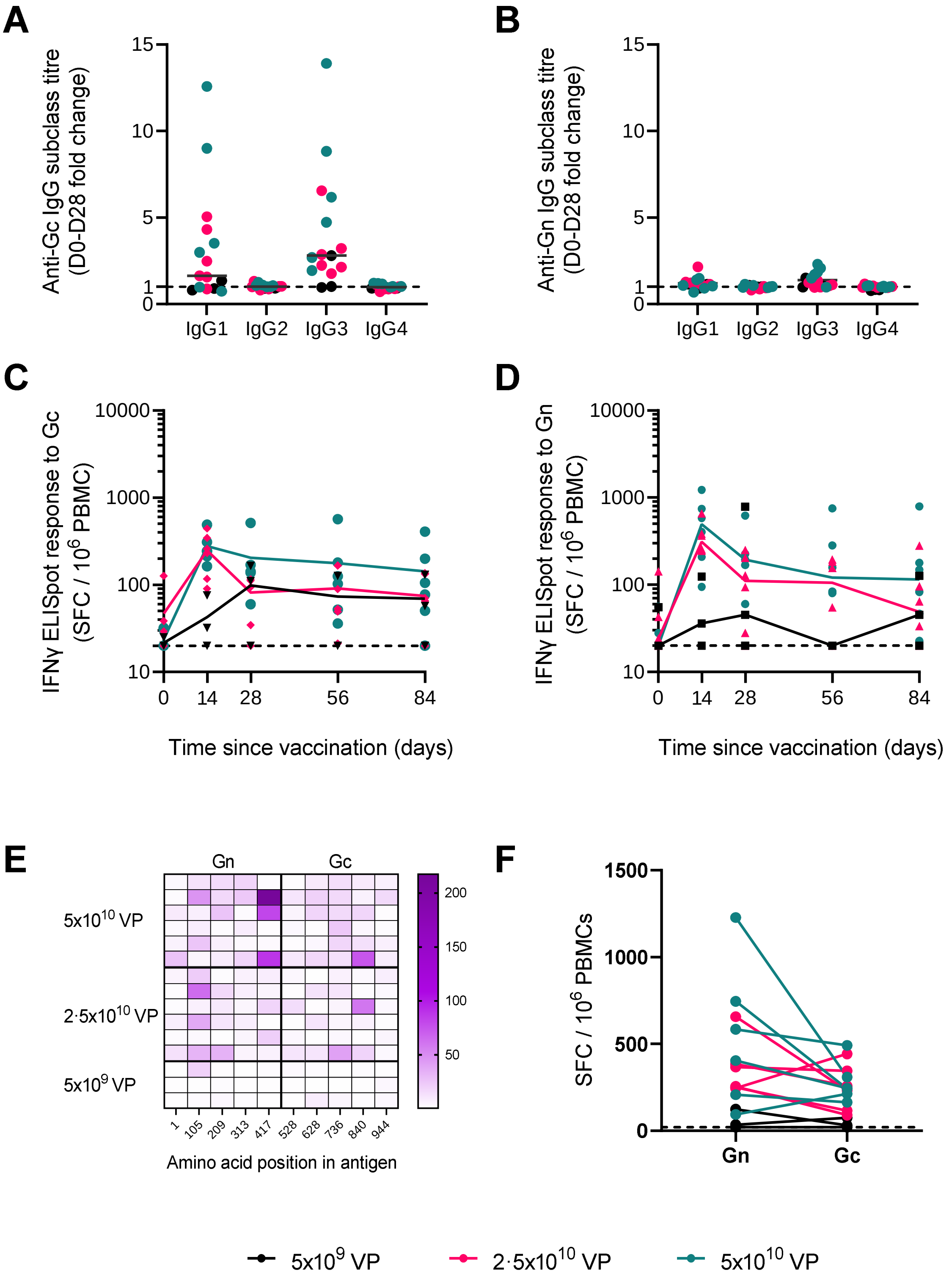

IgG subclass titre, plotted as fold change from baseline to day 28 post-vaccination towards A) Gc and B) Gn glycoprotein, respectively. Symbols represent mean values from two replicates. Horizontal lines represent median values. Summed IFNγ ELISpot response after PBMC stimulation with an overlapping peptide library spanning C) Gc and D) Gn glycoproteins, respectively. Symbols represent mean values from three replicates. Connecting lines represent median values. A heatmap of the IFN γ ELISpot response towards the peptide pools, visualising fold change from D0-D14 for each participant, is shown in (E). A comparison of the summed IFNγ ELISpot response to Gn versus Gc is shown in (F). Connecting lines represent data from the same individual**.**

Supplementary Figure 2: Correlations between immune parameters

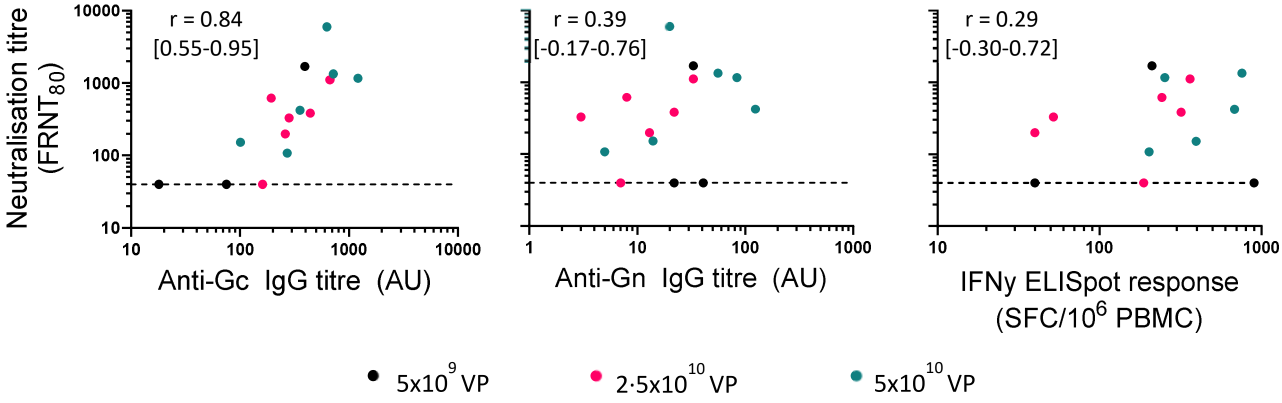

Correlations between volunteers' neutralising antibody titre and their (left) anti-Gc binding IgG titre, (middle) anti-Gn binding IgG titre and (right) IFNγ ELISpot response. Spearman correlations were used: $FRNT_{80}$ vs Gc ELISA r = 0.84 [95%CI 0.55-0.95]; $FRNT_{80}$ vs Gn ELISA, r = 0.39 [-0.17-0.76]; $FRNT_{80}$ vs IFNγ ELISpot response, r = 0.29 [-0.30-0.72]. Neutralisation titres, anti-Gc titres and anti-Gn IgG titres is from 28 days post-vaccination. IFN γ ELISpot data is from 14 days post-vaccination.

Supplementary Materials References

1. Wright D, Allen ER, Clark MHA, et al. Naturally Acquired Rift Valley Fever Virus Neutralizing Antibodies Predominantly Target the Gn Glycoprotein. *iScience* 2020; **23**(11): 101669.
2. Barrett JR, Belij-Rammerstorfer S, Dold C, et al. Phase 1/2 trial of SARS-CoV-2 vaccine ChAdOx1 nCoV-19 with a booster dose induces multifunctional antibody responses. *Nature medicine* 2021; **27**(2): 279-88.
3. Wichgers Schreur PJ, Paweska JT, Kant J, Kortekaas J. A novel highly sensitive, rapid and safe Rift Valley fever virus neutralization test. *Journal of virological methods* 2017; **248**: 26-30.
4. Kimani D, Jagne YJ, Cox M, et al. Translating the immunogenicity of prime-boost immunization with ChAd63 and MVA ME-TRAP from malaria naive to malaria-endemic populations. *Molecular therapy : the journal of the American Society of Gene Therapy* 2014; **22**(11): 1992-2003.

RVF001 Clinical Trial Protocol

UNIVERSITY OF OXFORD

A phase I study to determine the safety and immunogenicity of the candidate Rift Valley Fever Virus (RVFV) vaccine ChAdOx1 RVF in UK healthy adult volunteers

Study Reference: RVF001

Protocol Number: v3.0

Date: 27th Apr 2021

Chief Investigator: Professor A.V.S Hill

Sponsor: University of Oxford

Funder: UK BBSRC and MRC/Department of Health through the UK Vaccines Network

REC Number: 20/EE/0262

EudraCT Number: 2017-004482-27

IRAS Reference: 246707

©Oxford University

PAGE LEFT INTENTIONALLY BLANK

©Oxford University

**Full Study Title**	A phase I clinical trial to determine the safety and immunogenicity of the candidate RVFV vaccine ChAdOx1 RVF in UK healthy adult volunteers.<br>**Study Code**: RVF001<br>**EudraCT Number**: 2017-004482-27<br>**IRAS project ID**: 246707
**Short Study Title**	Safety and Immunogenicity of a candidate RVFV vaccine (RVF001)
**Chief Investigator**	**Prof Adrian V.S. Hill**<br>Centre for Clinical Vaccinology and Tropical Medicine<br>University of Oxford,<br>Churchill Hospital, Old Road, Headington<br>Oxford, OX3 7LE<br>Email: adrian.hill@ndm.ox.ac.uk
**Trial Sites**	**Centre for Clinical Vaccinology and Tropical Medicine**<br>Churchill Hospital, Old Road, Headington<br>Oxford, OX3 7LE
**Sponsoring Institution**	**University of Oxford**<br>Clinical Trials and Research Governance<br>Boundary Brook House<br>Churchill Drive<br>Headington<br>Oxford<br>OX3 7LQ<br>Tel: 01865 572224<br>Email: ctrg@admin.ox.ac.uk
**External Monitor**	**Clinical Trials & Research Governance**<br>Boundary Brook House<br>Churchill Drive<br>Headington<br>Oxford<br>OX3 7LQ

©Oxford University

Local Safety Monitor **Dr Brian Angus**
Centre for Clinical Vaccinology and Tropical Medicine
Churchill Hospital, Old Road, Headington
Oxford, OX3 7LE
[redacted]
Email: brian.angus@ndm.ox.ac.uk

©Oxford University

Confidentiality Statement

This document contains confidential information that must not be disclosed to anyone other than the Sponsor, the Investigator Team, HRA, host organisation, members of the Research Ethics Committee and other regulatory bodies. This information cannot be used for any purpose other than the evaluation or conduct of the clinical investigation without the prior written consent of Professor Adrian Hill.

Statement of Compliance

The trial will be conducted in compliance with the protocol, the principles Good Clinical Practice Guideline , Medicines for Human Use (Clinical Trial) Regulations 2004 (as amended) and all other applicable regulatory requirements.

Chief Investigator Approval and Agreement

I have read the trial protocol and agree to conduct the trial in compliance with the protocol, the principles of Good Clinical Practice and all applicable regulatory requirements.

I hereby approve this version of the protocol and declare no conflict of interest

Adrian Hill	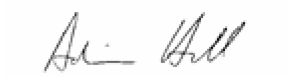	10/05/2021
**Chief Investigator Name**	**Signature**	**Date**

©Oxford University

Modification History

Version	Date	Author(s)	Modifications
1.0	6th Oct 2020	Pedro Folegatti, Daniel Jenkin, George Warimwe, Sarah Gilbert, Adrian Hill	N/A
1.1	8th Dec 2020	Daniel Jenkin, Nguyen Tran	Change of the planned trial period to Q1 2021 to Q4 2021.<br>Update the number of subjects exposed to ChAdOx1 vectored vaccines to date in section 3.8
2.0	6th Jan 2021	Daniel Jenkin, Pedro Folegatti, Nguyen Tran, Alison Lawrie	(Response to MHRA GNA)<br>Clarification of: staggered enrolment sequence, inclusion criteria related to pregnancy (updated to “at least 3 months”), temporary postponement of vaccination, sponsor responsibilities related to completion and submission of DSURs and group holding rules.<br>Update the list of abbreviations.
2.1	28th Jan 2021	Daniel Jenkin, Nguyen Tran	Section 6.3.1: Inclusion criterion 4 on contraception updated to “at least 3 months from the final vaccination procedure”
3.0	27th Apr 2021	Daniel Jenkin, Nguyen Tran, Alison Lawrie	Change of the planned trial period to Q2 2021 to Q4 2021.<br>Update to risks and exclusion criteria sections regarding reports of thrombosis and thrombocytopenia.

©Oxford University

Table of Contents

1. SYNOPSIS 10
2. ABBREVIATIONS 12
3. BACKGROUND & RATIONALE 14
3.1 Impact of Rift Valley Fever and the need for a vaccine 14
3.2 Progress towards a RVFV vaccine 15
3.3 Gn and Gc glycoproteins as a vaccine antigen 15
3.4 Adenovirus-vectored Vaccines 16
3.5 ChAdOx1 17
3.6 Development of ChAdOx1 RVF 17
3.7 Preclinical Studies 17
3.7.1 Efficacy and Immunogenicity 17
3.8 Previous clinical experience 21
3.9 Rationale 25
3.10 Vaccine Development Strategy 25
4. OBJECTIVES AND ENDPOINTS 26
4.1 Primary Objective 26
4.1.1 Primary Outcome Measures 26
4.2 Secondary Objective 26
4.2.1 Secondary Outcome Measures 26
5. STUDY OVERVIEW 28
5.1 Rationale for Selected Doses 28
5.2 Study Groups 29
5.2.1 Duration of study 29
5.2.2 Definition of Start and End of Trial 29
5.3 Potential Risks for volunteers 29
5.4 Known Potential Benefits 31
6. RECRUITMENT AND WITHDRAWAL OF TRIAL VOLUNTEERS 32
6.1 Volunteers 32
6.2 Informed consent 32
6.3 Inclusion and exclusion criteria 33
6.3.1 Inclusion Criteria 33
6.3.2 Exclusion Criteria 34
6.3.3 Effective contraception for female volunteers of childbearing potential 35
6.3.4 Prevention of 'Over Volunteering' 36
6.3.5 Criteria for postponement of vaccination 36
6.3.6 Withdrawal of Volunteers 36
6.4 Compliance with Dosing Regime 37
6.5 Pregnancy 37

©Oxford University

7. CLINICAL PROCEDURES 38
7.1 Study procedures 38
7.2 Observations 38
7.3 Blood Tests and Urinalysis 38
7.4 Study visits 39
7.4.1 Screening visit 39
7.4.2 Day 0: Enrolment and Vaccination Visit 40
7.4.3 Subsequent visits: days 2, 7, 14, 28, 56 and 84 42
7.4.4 COVID-19 Study Considerations 43
8. INVESTIGATIONAL PRODUCTS 45
8.1. Manufacturing and Presentation 45
8.1.1 Description of ChAdOx1 RVF 45
8.1.2 ChAdOx1 RVF formulation and packaging 45
8.2 Supply 45
8.3 Storage 45
8.4 Administration of Investigational Medicinal Products 45
8.5 Minimising environmental contamination with genetically modified organisms (GMO) 46
9. ASSESSMENT OF SAFETY 47
9.1 Definitions 47
9.1.1 Adverse Event (AE) 47
9.1.2 Adverse Reaction (AR) 47
9.1.3 Unexpected Adverse Reaction 47
9.1.4 Serious Adverse Event (SAE) 47
9.1.5 Serious Adverse Reaction (SAR) 48
9.1.6 Suspected Unexpected Serious Adverse Reaction (SUSAR) 48
9.2 Foreseeable Adverse Reactions: 48
9.3 Expected Serious Adverse Events 48
9.4 Causality Assessment 48
9.5 Reporting Procedures for All Adverse Events (see SOP VC027) 49
9.5.1 Reporting Procedures for Serious AEs (see SOP OVC005 Safety Reporting) 50
9.5.2 Reporting Procedures for SUSARS 50
9.5.3 Development Safety Update Report 50
9.6 Assessment of severity 50
9.7 Procedures to be followed in the event of abnormal findings 52
9.8 Local Safety Monitor 52
9.8.1 Interim Safety Reviews 53
9.9 Safety Stopping/Holding Rules 53
9.9.1 Group holding rules 53
10. STATISTICS 55

©Oxford University

11. DATA MANAGEMENT 56
11.1 Data Handling 56
11.2 Record Keeping 56
11.3 Source Data and Case Report Forms (CRFs) 56
11.4 Data Protection 57
11.5 Data Quality 57
11.6 Archiving 57
12. QUALITY CONTROL AND QUALITY ASSURANCE PROCEDURES 58
12.1 Investigator procedures 58
12.2 Monitoring 58
12.3 Protocol deviation 58
12.4 Audit & inspection 58
13. SERIOUS BREACHES 59
14. ETHICS AND REGULATORY CONSIDERATIONS 60
14.1 Declaration of Helsinki 60
14.2 Guidelines for Good Clinical Practice 60
14.3 Approvals 60
14.4 Volunteer Confidentiality 60
15. FINANCING AND INSURANCE 61
15.1 Financing 61
15.2 Insurance 61
15.3 Compensation 61
16. CONTRACTUAL ARRANGEMENTS 61
17. PUBLICATION POLICY 62
18. DEVELOPMENT OF A NEW PRODUCT/PROCESS OR THE GENERATION OF INTELLECTUAL PROPERTY 62
19. REFERENCES 63

©Oxford University

1. SYNOPSIS

**Trial Title**	A phase I study to determine the safety and immunogenicity of the candidate RVFV vaccine ChAdOx1 RVF in UK healthy volunteers.
**Trial Centre**	Centre for Clinical Vaccinology & Tropical Medicine, University of Oxford, Churchill Hospital, Old Road, Headington, Oxford, OX3 7LE
**Trial Identifier**	RVF001
**Clinical phase**	I
**Study Design**	Open–labelled, non-randomised, dose escalation, first-in-human, single centre, phase I clinical trial
**Population**	Healthy adults aged 18 – 50 years
**Planned Sample Size**	15 volunteers

Group	Dose of ChAdOx1 RVF
Group 1 (n=3)	5×10^{9} vp
Group 2 (n=6)	2.5×10^{10} vp
Group 3 (n=6)	5×10^{10} vp

**Follow-up duration**	12 weeks post vaccine administration
**Planned Trial Period**	Q2 2021 to Q4 2021
**Primary Objective**	To assess the safety profile of the candidate vaccine ChAdOx1 RVF in healthy adult volunteers
**Secondary Objective**	To assess the immunogenicity of the candidate vaccine ChAdOx1 RVF in healthy adult volunteers

©Oxford University

**Investigational Products**	ChAdOx1 RVF, a replication-deficient simian adenoviral vector encoding the envelope glycoproteins (Gn/Gc) of the Rift Valley Fever Virus.
**Dose per Administration**	ChAdOx1 RVF 5 x 10^9 vp<br>ChAdOx1 RVF 2.5 x 10^{10} vp<br>ChAdOx1 RVF 5 x 10^{10} vp
**Form**	Liquid (all finished products)
**Route**	Intramuscularly (IM) into the deltoid region of the arm

©Oxford University

2. ABBREVIATIONS

AE	Adverse event
AR	Adverse reaction
CBF	Clinical Biomanufacturing Facility
CCVTM	Centre for Clinical Vaccinology and Tropical Medicine
ChAdOx1	Chimpanzee Adenovirus Ox1
ChAdOx1 RVF	Recombinant Chimpanzee Adenovirus Ox1 encoding the envelope glycoproteins of RVFV
CI	Chief Investigator
CRF	Case Report Form or Clinical Research Facility
CTRG	Clinical Trials Research Governance
DSUR	Development Safety Update Report
EC	Ethics committee
ELISA	Enzyme linked immunosorbent assay
ELISpot	Enzyme linked immunospot assay
FBC	Full blood count
GCP	Good Clinical Practice
GMO	Genetically modified organism
GMP	Good Manufacturing Practice
HBsAg	Hepatitis B surface antigen
HCG	Human Chorionic Gonadotrophin
HCV	Hepatitis C virus
HIV	Human Immunodeficiency virus
HLA	Human leukocyte antigen
IB	Investigators Brochure
ICH	International Conference on Harmonisation
IM	Intramuscular
IMP	Investigational Medicinal Product
LSM	Local Safety Monitor
MHRA	Medicines and Healthcare products Regulatory Agency
MVA	Modified Vaccinia Virus Ankara
OIE	World Organisation for Animal Health
PFU	Plaque Forming Units
PIS	Participant information sheet
QP	Qualified Person
RVF	Rift Valley Fever
RVFV	Rift Valley Fever Virus

©Oxford University

SAE	Serious Adverse Event
SAR	Serious Adverse Reaction
SFU	Spot forming units
SOP	Standard Operating Procedure
SUSAR	Suspected Unexpected Serious Adverse Reaction
TMF	Trial Master File
vp	Viral particles
WHO	World Health Organisation

©Oxford University

3. BACKGROUND & RATIONALE

3.1 Impact of Rift Valley Fever and the need for a vaccine

Rift Valley Fever Virus (RVFV) is a negative-stranded RNA *Phlebovirus* in the *Bunyaviridae* family known to be the causative agent of Rift Valley Fever (RVF), an emerging mosquito-borne zoonotic infectious viral disease first described in the early 1930s (1). RVF affects primarily ruminants and it is characterised by high mortality rates (>90%) amongst young sheep, goats and cattle and increased abortion episodes in pregnant animals (2). The disease has serious implications for livestock agriculture and trade and is listed as a notifiable disease by the World Organization for Animal Health (OIE). Although primarily restricted to Africa, the virus can be transmitted by numerous different mosquito species that are more widely distributed than RVFV itself (1), leading to concerns of disease spread as has occurred in the Arabian Peninsula and Madagascar (3, 4). Humans can get infected through either infectious mosquito bites or contact with virus-contaminated tissues and fluid (5, 6). In humans, RVFV infection usually presents as an acute self-limiting febrile illness, but severe manifestations including haemorrhagic fever and encephalitis may also occur. Previous outbreaks in East Africa and Saudi Arabia reported case fatality rates of 30% and long-term sequela (e.g. impaired vision) were observed in some of the survivors (7, 8).

Outbreaks of the disease are episodic and closely linked to climate variability, especially prolonged heavy rains and flooding that facilitates RVFV transmission by vector mosquitoes (9). It is thought that successive and overlapping swarms of different mosquitos infect and increase infection rates in ruminants with subsequent transmission to humans, resulting in epidemics (10). Human epidemics during RVF outbreaks in eastern Africa (Kenya, Tanzania, Somalia, Sudan and Madagascar) involved 478 deaths in 1998, 1107 reported cases with 350 deaths in 2006 and 2007, and 1174 cases with 241 deaths in 2008 (11). More recently, the Ministry of Health of Niger reported 105 suspected cases in 2016, including 28 deaths of RVF in humans in the Tahoua region (12), and an RVF outbreak is currently ongoing in South Sudan. The high levels of human morbidity and mortality during these outbreaks underscores the urgency of developing comprehensive surveillance, response and control programmes, especially since there is growing evidence for inter-epidemic transmission of RVFV (13, 14). In Uganda, three RVF outbreaks have been reported; one in 1968 (15), 2016 (16), and there is currently an ongoing outbreak reported since November 2017 (17). There is also evidence of RVF endemicity on livestock farms in Uganda (18), Kenya, Rwanda, and other countries in eastern Africa.

RVFV is an emerging global health threat listed as a select agent with significant potential for international spread and use in bioterrorism, hence it has been prioritised for vaccine research and development by the World Health Organisation (WHO), the National Institute of Allergy and Infectious Diseases (NIAID) and the UK Vaccine Research & Development Network (UKVRDN) (19). Live and inactivated RVFV vaccines are available for livestock, but no licensed vaccines or anti-viral therapies are currently available for humans (20).

©Oxford University

3.2 Progress towards a RVFV vaccine

Vaccination continues to be the most effective way to control RVF (21). The widely used live attenuated RVFV vaccines (e.g. Smithburn vaccine) in livestock elicit high titre neutralizing antibody and provide durable cross-strain protection after a single vaccination and have therefore been valuable for the control of RVFV in endemic areas. However, vaccination of pregnant animals can result in abortions and fetal malformations due to residual virulence, making their general use in humans very unlikely (21, 22). A formalin-inactivated investigational RVFV vaccine, TSI-GSD-200, has previously been evaluated in humans and found to be safe but poorly immunogenic, requiring three primary immunizations and a booster dose to generate and maintain neutralizing antibody responses (23, 24). An investigational live RVFV vaccine, MP-12, has shown better immunogenicity in humans than TSI-GSD-200, but as with the vaccine used in livestock, it could lead to post-vaccination viremia potentially causing teratogenic effects or abortion (25).

Development and licensure of safe, highly immunogenic and efficacious RVFV vaccines for humans is clearly an unmet need for this neglected public health threat. The use of vaccine platforms with a well-established human safety profile is an attractive strategy for this purpose (20). Recovery from natural RVFV infection results in long-lived cross-strain immunity conferred by neutralizing antibodies against the viral envelope glycoproteins, Gn and Gc, which are conserved across different viral isolates (26, 27). Thus, whilst the neutralizing antibody titre threshold required for protection against RVFV infection is currently unknown, development of vaccines that elicit antibody titres within the range induced by natural infections is a very attractive way forward.

3.3 Gn and Gc glycoproteins as a vaccine antigen

Bunyaviruses are enveloped viruses which harbor a tripartite, single stranded RNA genome with negative polarity. The tri-partite viral genome consists of a small (S), medium (M) and large (L) RNA genome segments. The L-segment of the genome encodes for the viral polymerase, the M-segment for the viral glycoproteins, Gn and Gc, and the S-segment for the nucleocapsid (N) protein (28).

The Gn and Gc are the two major structural glycoproteins involved in virus–cell attachment and membrane fusion and are the only targets for neutralizing antibodies (29). Phleboviruses use their envelope proteins, Gn and Gc, for entry into target cells and for assembly of progeny particles in infected cells. Binding of Gn and Gc to cell surface factors promotes viral attachment and uptake into cells, and exposure to endosomal low pH induces Gc-driven fusion of the viral and the vesicle membranes. Moreover, Gn and Gc facilitate virion incorporation of the viral genome via their intracellular domains and Gn and Gc interactions allow the formation of a highly ordered glycoprotein lattice on the virion surface (28).

Gn and Gc are synthesized as a precursor polyprotein, GnGc, in the secretory pathway of infected cells. Gn and Gc are separated by proteolytic cleavage but may remain non-

©Oxford University

covalently associated. The cleavage step is executed by a cellular enzyme, signal peptidase, during import of the GnGc precursor into the endoplasmic reticulum (ER). In the ER, Gn and Gc are decorated with N-linked glycans of the high-mannose type, which can be processed into hybrid and complex forms upon import of Gn and Gc into the Golgi apparatus. The Golgi apparatus is the site of bunyavirus budding and this process is facilitated by Gn and Gc, which play a key role in particle morphogenesis and genome incorporation. Finally, infectious particles decorated with Gn and Gc are released from the infected cell by exocytosis (28).

The roles of Gn and Gc in viral entry into host cells and membrane fusion make them a perfect target for vaccine development.

3.4 Adenovirus-vectored Vaccines

Adenoviruses are attractive vectors for human vaccination. They possess a stable genome so that inserts of foreign genes are not deleted and they can infect large numbers of cells without any evidence of insertional mutagenesis.

Replication defective adenovirus can be engineered by deletion of genes from the E1 locus, which is required for viral replication, and these viruses can be propagated easily with good yields in cell lines expressing E1 from AdHu5 such as human embryonic kidney cells 293 (HEK 293 cells) (30).

Previous mass vaccination campaigns in over 2 million adult US military personnel using orally administered live human adenovirus serotype 4 and 7 have shown good safety and efficacy data (31). Human adenoviruses are under development as vectors for malaria, HIV, tuberculosis and hepatitis C vaccines, amongst others. They have been used extensively in human trials (32-36) with excellent safety profile mainly as vectors for HIV vaccines.

A limiting factor to widespread use of human adenovirus as vaccine vectors has been the level of anti-vector immunity present in humans where adenovirus is a ubiquitous infection. The prevalence of immunity to human adenoviruses prompted the consideration of simian adenoviruses as vectors, as they exhibit hexon structures homologous to human adenoviruses (37). Simian adenoviruses are not known to cause pathological illness in humans and the prevalence of antibodies to chimpanzee origin adenoviruses is less than 5% in humans residing in the US (38).

In chimpanzee adenoviruses, the E1 locus can be deleted to render viruses replication deficient and allow transcomplementation on an E1 AdHu5 complementing cell line (39). Whilst they exhibit hexon structures homologous to that of human adenoviruses (40), the lack of sequence homology at the E1 flanking sequence prevents homologous recombination and production of replication competent virus (41)

Chimpanzee adenoviral vectors can be manufactured cost-effectively and are now in clinical development as possible vaccines against malaria, HIV, tuberculosis, influenza, hepatitis C, RSV, Cancer and Ebola (42-47).

©Oxford University

3.5 ChAdOx1

ChAdOx1 is a novel recombinant chimpanzee adenovirus designed as a vaccine vector, developed by The Jenner Institute at the University of Oxford. This viral vector has been used by researchers at the University of Oxford to produce a number of vaccines expressing a range of different antigens.

ChAdOx1 is produced from a replication-deficient (E1 and E3 deleted) simian adenovirus and it has been described by Dicks et al (48). The vector was constructed in a bacterial artificial chromosome (BAC) to facilitate genetic manipulation of genomic clones with improved stability and flexibility. Cellular immunogenicity of recombinant E1 E3-deleted ChAdOx1 was comparable to that of other species E derived chimpanzee adenovirus vectors including ChAd63, the first simian adenovirus vector to enter clinical trials in humans. The E1 region is essential for viral replication, hence the ability to delete E1 renders the new vector immediately replication incompetent. The deletion of the non-essential adenovirus E3 region increases the insert capacity of the new vector by approximately 5kb. It is known that the proteins encoded by the E4 region of adenoviruses interact with E1 during viral replication, and the imperfect interaction between the gene products of the AdHu5 E1 gene produced by HEK293 cells and simian E4 gene products has been found to result in impaired viral replication in this cell line, and consequently lower virus yields. In ChAdOx1, Ad5 E4Orf4 has been inserted to replace the homologous simian virus coding sequence, resulting in improved viral replication during vaccine production. Since no replication of the virus takes place after immunization, this replacement has no effect on immunogenicity of the viral vector. Insertion of recombinant antigens at the E1 locus is performed using Gateway® site specific recombination technology (Invitrogen).

3.6 Development of ChAdOx1 RVF

ChAdOx1 RVF encodes the RVFV envelope glycoproteins that are antigenically conserved targets of protective neutralising antibodies. A genomic clone of ChAdOx1 RVF was prepared by Gateway® recombination between an entry plasmid containing the coding sequence for RVFV Gn and Gc (Genbank accession number DQ380208, bases 411–3614) and the E1-and E3-deleted ChAdOx1 destination vector. The vector encoded the 1,067 amino acid Gn and Gc RVF viral polyprotein under the control of the human cytomegalovirus major immediate early promoter.

3.7 Preclinical Studies

3.7.1 Efficacy and Immunogenicity

An initial efficacy and immunogenicity study of ChAdOx1 RVF has been conducted previously in mice (49). A single-dose immunisation with ChAdOx1 RVF was shown to elicit high-titre neutralizing antibodies and confer protection against a lethal viral challenge eight weeks post-immunization. The vaccine also elicited T cells responses assessed by interferon gamma (IFNγ) production by splenocytes obtained from mice as measured by enzyme-linked immunospot

©Oxford University

assay (ELISpot). Intracellular cytokine staining assay of peripheral blood mononuclear cells (PBMCs) sampled two weeks post-vaccination revealed a robust CD8+ T cell response mainly comprising cells staining positive for IFNγ or tumor necrosis factor alpha (TNFα) or both these cytokines (49).

The vaccine has been further tested in target animals in disease-endemic settings in Kenya (sheep, goats and cattle) and Saudi Arabia (dromedary camels) (50). Immunogenicity and protective efficacy against RVFV challenge were the primary endpoints. Sheep, goats and cattle aged 4–6 months were sourced from local commercial farms in Kenya and pre-screened for previous exposure to RVFV. Groups of seronegative animals were then immunized intramuscularly with 10^9 infectious units (iu) ChAdOx1 RVF alone (group 1), 10^9 iu ChAdOx1 RVF mixed with the saponin-based Matrix-Q™ adjuvant (group 2) or placebo comprising phosphate buffered saline (PBS; group 3). A fourth comparator group received the licensed live Smithburn RVFV livestock vaccine widely used in Africa (group 4). Four weeks later all animals were challenged by subcutaneous inoculation of 10^7 plaque-forming units (pfu) of the same batch of a heterologous virulent RVFV strain and monitored for 14 days after which they were culled. The primary endpoint for efficacy was development of viraemia as measured by qRT-PCR in whole blood sampled daily over the monitoring period. All sheep, goats and cattle in the placebo group developed viraemia. No viraemia could be detected among any of the ChAdOx1 RVF vaccinees in all three species (Fig. 1). The licensed Smithburn vaccine was also protective, but one goat and one calf receiving this vaccine developed viraemia post-challenge. In all three species ChAdOx1 RVF vaccination elicited high-titre neutralizing antibody, comparable to the licensed Smithburn vaccine (Fig. 2a). Similar neutralizing antibody titres were observed in parallel ChAdOx1 RVF immunogenicity studies in sheep and cattle in the UK, and remained high at 3 months post-vaccination (51). Matrix-Q™ adjuvant had no effect on the antibody response elicited by ChAdOx1 RVF in any of the livestock species (Fig. 2a), as opposed to what was previously seen in mice (52), highlighting the importance of evaluating candidate vaccine regimens in target species. Significant post-challenge boosting of neutralizing antibody titres was observed in all three species independent of vaccine allocation (Fig. 2c) (20).

©Oxford University

[redacted]

Figure 1. ChAdOx1 RVF vaccination protects sheep, goats and cattle against RVFV challenge. Kaplan-Meier plots are used to infer vaccine-mediated protection using the primary endpoint of qRT-PCR detection of viraemia over a 14-day period following challenge (a, sheep; d, goats;

©Oxford University

g, cattle). Peak viraemia levels for each species are shown as relative plaque-forming units (pfu; bars represent means), estimated by extrapolation from a standard curve generated using serial dilutions of RNA isolated from the challenge virus and assayed using the same method as the test samples (b, sheep; e, goats; h, cattle). Rectal temperature data measured at the same time of day during post-challenge monitoring are shown by vaccine allocation (c, sheep; f, goats; i, cattle), presented as means and standard errors (20).

Six of seven camels immunised with ChAdOx1 RVF in Saudi Arabia mounted a neutralizing antibody response detectable at day 28 post-vaccination (Fig. 2b) and the remaining ChAdOx1 RVF vaccinee had seroconverted by the next sampling time point (day 56 post-vaccination) (20).

[redacted]

©Oxford University

Figure 2. ChAdOx1 RVF elicits high-titre RVFV neutralizing antibody. For each species means and standard errors of RVFV neutralizing antibody titres measured 28 days post-vaccination are shown in (a) and titres measured 14 days post-challenge are shown in (c). Pooled pre-challenge (b) and post-challenge (d) neutralizing antibody data from vaccinees (ChAdOx1 RVF groups and Smithburn group) are shown, with each point representing an animal and bars representing the means and standard errors. Neutralizing antibody titres measured 28 days post-ChAdOx1 RVF immunization in dromedary camels are shown in (b). All analyses are by the Kruskal-Wallis test, with Dunn's correction for multiple comparisons between groups. ns=not statistically significant ($p > 0.05$). The dashed line represents the detection limit of the assay (20).

3.8 Previous clinical experience

This will be the first-in-human study using ChAdOx1 RVF. However, ChAdOx1 vectored vaccines expressing different inserts have previously been used over 8000 healthy volunteers taking part in clinical trials conducted by the University of Oxford in the UK and overseas. ChAdOx1 vectored vaccines encoding antigens from Influenza, Tuberculosis, Prostate Cancer, Malaria, MERS-CoV, Chikungunya, Meningitis B and Zika have entered clinical testing as part of multiple phase I clinical trials, as summarised in table 1.

None of the studies mentioned reported serious adverse events associated with the administration of ChAdOx1, which was shown to have a good safety profile.

©Oxford University

Table 1. Clinical experience with ChAdOx1 viral vector vaccines.

Country	Trial	Vaccine	Age	Route	Dose	Number of Volunteers (Received ChAdOx1)	Publication / Registration Number
UK	FLU004	ChAdOx1 NP+M1	18-50	IM	$5x10^8$ vp	3	Antrobus et al, 2014. Molecular Therapy. DOI: 10.1038/mt.2013.284
					$5x10^9$ vp	3	
					$2.5x10^{10}$ vp	3	
					$5x10^{10}$ vp	6	
UK	FLU005	ChAdOx1 NP+M1 MVA NP+M1 (week 8)	18-50	IM	$2.5x10^{10}$ vp	12	Coughlan et al, 2018. EBioMedicine DOI: 10.1016/j.ebiom.2018.02.011 DOI: 10.1016/j.ebiom.2018.05.001
		ChAdOx1 NP+M1 MVA NP+M1 (week 52)	18-50	IM	$2.5x10^{10}$ vp	12	
		MVA NP+M1 ChAdOx1 NP+M1 (week 8)	18-50	IM	$2.5x10^{10}$ vp	12	
		MVA NP+M1 ChAdOx1 NP+M1 (week 52)	18-50	IM	$2.5x10^{10}$ vp	9	
		ChAdOx1 NP+M1	>50	IM	$2.5x10^{10}$ vp	12	
		ChAdOx1 NP+M1 MVA NP+M1 (week 8)	>50	IM	$2.5x10^{10}$ vp	12	
UK	TB034	ChAdOx1 85A	18-50	IM	$5x10^9$ vp	6	Wilkie et al, 2020 Vaccine DOI: 10.1016/j.vaccine.2019.10.102
					$2.5x10^{10}$ vp	12	
		ChAdOx1 85A MVA85A (week 8)	18-50	IM	$2.5x10^{10}$ vp	12	
		ChAdOx1 85A (x2, 4weeks apart) MVA85A (at 4 months)	18-50	IM	$2.5x10^{10}$ vp	12	
Switzerland	TB039 (ongoing)	ChAdOx1 85A	18-55	Aerosol	$1x10^9$ vp	3	Clinicaltrials.gov: NCT04121494
				Aerosol	$5x10^9$ vp	3	
				Aerosol	$1x10^{10}$ vp	11	
				Aerosol/IM	$1x10^{10}$ vp	15	
Uganda		ChAdOx1 85A	18-49	IM	$5x10^9$ vp	6	Clinicaltrials.gov:

©Oxford University

Country	Trial	Vaccine	Age	Route	Dose	Number of Volunteers (Received ChAdOx1)	Publication / Registration Number
	TB042 (ongoing)				2.5 x10^{10}	6	NCT03681860
UK	VANCE01	ChAdOx1.5T4 MVA.5T4	18 – 75	IM	2.5x10^{10} vp	34	Clinicaltrials.gov: NCT02390063
UK	ADVANCE (ongoing)	ChAdOx1.5T4 MVA.5T4	≥18	IM	2.5x10^{10} vp	23 (as of Feb 20)	Clinicaltrials.gov: NCT03815942
UK	VAC067	ChAdOx1 LS2	18-45	IM	5x10^{9} vp	3	Clinicaltrials.gov: NCT03203421
					2.5x10^{10} vp	10	
UK	MERS001	ChAdOx1 MERS	18-50	IM	5x10^{9} vp	6	Clinicaltrials.gov: NCT03399578 DOI: https://doi.org/10.4269/ajtmh.abstract2018 Abstract#973, page 305.
					2.5x10^{10} vp	9	
					5x10^{10} vp	9	
					2.5x10^{10} vp (homologous prime-boost)	2	
UK	VAMBOX	ChAdOx1 MenB.1	18-50	IM	2.5x10^{10} vp	3	ISRCTN46336916
					5x10^{10} vp	26	
UK	CHIK001	ChAdOx1 Chik	18-50	IM	5x10^{9} vp	6	Clinicaltrials.gov: NCT03590392 DOI: https://doi.org/10.4269/ajtmh.abstract2019 Abstract #59, page 19.
					2.5x10^{10} vp	9	
					5x10^{10} vp	9	
UK	ZIKA001	ChAdOx1 Zika	18-50	IM	5x10^{9} vp	6	Clinicaltrials.gov: NCT04015648
					2.5x10^{10} vp	9 (as of May 20)	
					5x10^{10} vp	-	
UK	COV001	ChAdOx1 nCoV-19	18-55	IM	5 × 10^{10} vp	544	Folegatti, Ewer et al. Lancet 2020 (53)

©Oxford University

Country	Trial	Vaccine	Age	Route	Dose	Number of Volunteers (Received ChAdOx1)	Publication / Registration Number
	(ongoing)						Clinicaltrials.gov: NCT04324606
UK	COV002 (ongoing)	ChAdOx1 nCoV-19	18-55	IM	5×10^{10} vp	4138	Clinicaltrials.gov: NCT04400838
			56-69		5×10^{10} vp	149	
			≥70		5×10^{10} vp	186	
Brazil	COV003 (ongoing)	ChAdOx1 nCoV-19	18-55	IM	5×10^{10} vp	2019 (as of Aug 2020)	ISRCTN89951424
South Africa	COV005 (ongoing)	ChAdOx1 nCoV-19	18-65	IM	5×10^{10} vp	698 (as of Aug 2020)	Clinicaltrials.gov: NCT04444674

©Oxford University

3.9 Rationale

RVFV is an emerging zoonotic mosquito-borne viral disease with serious implications for livestock agriculture and trade, leading to significant economic losses and social impact. Furthermore, the virus is considered a global threat and it has been listed as a select agent with significant potential for international spread and use in bioterrorism. Previous RVF outbreaks in Somalia (2006-2007), Kenya (2006-2007), Tanzania (2007), and Sudan (2007-2008) showed extensions to districts, which were not involved before. These outbreaks also demonstrated the changing epidemiology of the disease from being originally associated with livestock, to a seemingly highly virulent form infecting humans and causing considerably high-fatality rates (54). More recently, RVFV has been associated with miscarriages in pregnant women (55).

The widely used live attenuated RVFV vaccines in livestock in Africa are considered to be highly effective, but major safety concerns preclude their general use in humans. There are no licensed vaccines currently available for human use.

Chimpanzee adenovirus vaccine vectors have been safely administered to over thousands of people using a wide range of infectious disease targets including malaria (56), HIV (57), tuberculosis, influenza (58), hepatitis C (59), RSV (60) and, most recently, Ebola (61). ChAdOx1 viral vectored vaccines have shown to be both safe and immunogenic in previous clinical trials in the UK and a single-dose immunisation with ChAdOx1 RVF vaccine has shown to elicit high levels of neutralising antibody in target animal species (sheep, goats and cattle). These neutralising antibody titres are comparable to those elicited by the most immunogenic licensed livestock vaccine (i.e. Smithburn vaccine) and are within the range observed in naturally exposed humans living in RVFV-endemic settings in East Africa (20)(Warimwe, unpublished observations). These data have underpinned the co-development of ChAdOx1 RVF for use in both livestock and humans.

3.10 Vaccine Development Strategy

A phase I clinical trial at a disease-endemic area (the MRC/Uganda Virus Research Unit, Entebbe) is planned following the ChAdOx1 RVF trial in Oxford. The optimal dose of ChAdOx1 RVF will be determined following the Oxford and Ugandan trials and, provided there are no safety concerns, phase II studies will be conducted in Uganda and Kenya.

©Oxford University

4. OBJECTIVES AND ENDPOINTS

The number of volunteers has been chosen to generate adequate safety and immunogenicity data to meet these objectives, whilst minimising the number of volunteers exposed to a new vaccination regimen.

4.1 Primary Objective

To assess the safety and tolerability of ChAdOx1 RVF in healthy adult volunteers.

4.1.1 Primary Outcome Measures

The specific endpoints for safety and reactogenicity will be actively and passively collected data on adverse events.

The following parameters will be assessed for all study groups

- Occurrence of solicited local reactogenicity signs and symptoms for 7 days following the vaccination
- Occurrence of solicited systemic reactogenicity signs and symptoms for 7 days following the vaccination
- Occurrence of unsolicited adverse events for 28 days following the vaccination
- Change from baseline for safety laboratory measures
- Occurrence of serious adverse events during the whole study duration

Volunteers will undergo clinical follow up for a further 90 days following completion of the vaccination regimen. SAEs will be collected throughout the study. The duration of follow up reflects the desire to obtain safety data with the first use of ChAdOx1 RVF in humans.

4.2 Secondary Objective

To assess the cellular and humoral immunogenicity of ChAdOx1 RVF in healthy adult volunteers.

4.2.1 Secondary Outcome Measures

Measures of immunogenicity to the ChAdOx1 RVF vaccine may include:

- ELISA to quantify antibodies to GnGc proteins
- RVFV neutralising antibody titres
- Ex vivo ELISpot and flow cytometry responses to GnGc

Other exploratory immunology may be carried out in collaboration with other specialist laboratories, including laboratories outside of Europe. This would involve transfer of serum/plasma and/or peripheral blood

©Oxford University

mononuclear cells (PBMC), but samples would be anonymised. Volunteers will be asked for their consent for this before enrolment.

©Oxford University

5. STUDY OVERVIEW

This is a first-in-human, open-label, dose escalation, phase I clinical trial to assess the safety and immunogenicity of the candidate ChAdOx1 RVF vaccine in healthy volunteers aged 18-50. The vaccine will be administered intramuscularly.

Volunteers will be recruited and vaccinated at the CCVTM, Oxford. There will be 3 study groups and a total of 15 volunteers will be enrolled (table 2). The sequence of enrolment is described in section 7.4.2.2. Volunteers will be initially recruited into Group 1 and subsequently into Groups 2 and 3 following interim clinical safety reviews (see section 7.4.2). Volunteers will be allocated to a study group by selecting eligible volunteers for enrolment following screening.

5.1 Rationale for Selected Doses

Doses to be administered in this trial have been selected on the basis of clinical experience with the ChAdOx1 adenovirus vector expressing different inserts and similar adenovirus vectored vaccines (eg. ChAd63).

A first-in-human dose escalation study using the ChAdOx1 vector encoding an influenza antigen (FLU004), safely administered ChAdOx1 NP+M1 at doses ranging from 5×10^8 to 5×10^{10} vp. Subsequent review of the data identified an optimal dose of 2.5×10^{10} vp balancing immunogenicity and reactogenicity. This dose has subsequently been given to over 175 volunteers in numerous larger phase 1 studies at the Jenner Institute (FLU005, TB034 VANCE01, ADVANCE and VAC067) and ChAdOx1 vectored vaccines have thus far demonstrated to be very well tolerated. The vast majority of AEs have been mild-moderate and there have been no SARs until this date in these studies.

Other ChAdOx1 vectored vaccines have been given at doses up to 5×10^{10}vp (MERS001, VAMBOX, CHIK001 and nCoV-19) whilst a different simian adenovirus vector (ChAd63) has been safely administered at doses up to 2×10^{11} vp with an optimal dose of 5×10^{10} vp, balancing immunogenicity and reactogenicity.

As this is a first-in-human assessment of the RVFV GnGc antigenic insert, the first dose of ChAdOx1 RVF proposed in this study (5×10^9 vp) is therefore 10 fold less than the expected tolerable dose of ChAdOx1 RVF (5×10^{10} vp). Doses will be gradually increased aiming to provide an optimal dose of ChAdOx1 RVF considering the tolerability, reactogenicity and immunogenicity profiles.

©Oxford University

5.2 Study Groups

Table 2. Study Groups

Group	Single Dose ChAdOx1 RVF	Route
Group 1 (n=3)	5×10^{9} vp	IM
Group 2 (n=6)	2.5×10^{10} vp	IM
Group 3 (n=6)	5×10^{10} vp	IM

5.2.1 Duration of study

The total duration of the study will be 12 weeks from the day of enrolment for all volunteers.

5.2.2 Definition of Start and End of Trial

The start of the trial is defined as the date of the first vaccination of the first volunteer. The end of the trial is the date of the last visit of the last volunteer.

5.3 Potential Risks for volunteers

The potential risk to participants is considered as low. The potential risks are those associated with phlebotomy and vaccination. In general, recombinant adenoviral vectors are safe. Similar vaccines encoding different antigens have been given to several thousand volunteers (including children) with a good safety profile.

Phlebotomy:

The maximum volume of blood drawn over the study period (approximately 300mL) should not compromise these otherwise healthy volunteers. There may be minor bruising, local tenderness or pre-syncopal symptoms associated with venepuncture, which will not be documented as AEs if they occur.

Vaccination:

ChAdOx1 RVF has not been used in humans before and therefore will be initially administered at the lower dose of 5×10^{9} vp before progressing to the higher doses of 2.5×10^{10} and 5×10^{10} vp in Groups 2 and 3. Expected risks from vaccination include local effects such as pain, redness, warmth, swelling, tenderness or itching. Systemic reactions that could potentially occur following immunisation with a recombinant adenovirus vaccine include a flu-like illness with feverishness, fatigue, malaise, arthralgia, myalgia and headache.

As with any vaccine, Guillain-Barré syndrome or immune-mediated reactions that can lead to organ damage may occur, but this should be extremely rare. Serious allergic reactions including anaphylaxis could also occur and for this reason volunteers will be vaccinated in a

©Oxford University

clinical area where Advanced Life Support trained physicians, equipment and drugs are immediately available for the management of any serious adverse reactions (SAR).

Thrombosis with Thrombocytopenia:

In April 2021, MHRA and JCVI conducted a review of extremely rare reports of cerebral venous sinus thrombosis (and thrombosis of other major veins) with concurrent thrombocytopenia occurring after vaccination with ChAdOx1 nCoV-19 in the national COVID-19 vaccination programme. The potential mechanism of these events, and whether they are related to the ChAdOx1 viral vector or to the SARS-CoV-2 spike protein, is yet to be determined. Estimates of incidence of thrombosis with thrombocytopenia following ChAdOx1 nCoV-19 vaccination vary and may change further as more cases are gathered and case definitions are finalised; current UK estimates are of cases occurring after less than 1 in 100,000 prime vaccinations.

On the basis of their ongoing scientific review the UK Medicines Healthcare Regulatory Agency have concluded: "the evidence of a link with vaccination and COVID-19 is stronger, but more work is still needed"(62). The European Medicines Agency concluded that unusual blood clots with low blood platelets should be listed as very rare side effects of this vaccine. Both agencies concluded that there wasn't enough evidence at present to say what the risk factors (e.g. age, gender, or other medical conditions) might be for having one of these rare clotting problems. Although the MHRA have received a higher number of reports from younger adults than older individuals. Cases of thrombosis with thrombocytopenia have also been reported after COVID-19 Vaccine Janssen, an adenovirus serotype 26 vectored spike-protein based COVID-19 vaccine. The EMA has also conducted a review of these cases and concluded there is a likely causal relationship with the Janssen vaccine(63).

Thrombosis with thrombocytopenia syndrome has not previously been reported following the use of live oral adenovirus serotype 4 and serotype 7 adenovirus vaccines in the United States, where these vaccines have been approved for vaccination of military personel(64-66). Over 1.3 million doses have been administered between 2011 to 2018 with no reports of thrombosis following vaccination to the US CDC/FDA Vaccine Adverse Event Reporting System (VAERS) post-marketing passive vaccine safety monitoring database(66).

Both the MHRA and EMA have confirmed that the evidence to date does not suggest that the COVID-19 Vaccine AstraZeneca causes venous thromboembolism *without* a low platelet count(62, 67).

All volunteers will be provided with this information via the study information booklet and will be kept updated should further information become available. They will also be specifically counselled on symptoms that could be warning signs of serious clotting events, as per public health advice being given to people receiving ChAdOx1 nCoV-19.

©Oxford University

Potential interaction with other adenoviral vectored vaccines:

The ChAdOx1 vector used in the ChAdOx1 RVF experimental vaccine is the same as that used in the Oxford AstraZeneca and similar to the one used in the Janssen (Johnson and Johnson) COVID-19 vaccines (Adenovirus 26). There is a theoretical risk that receiving the experimental ChAdOX1 RVF vaccine may reduce the benefit of subsequent administrations of certain vaccines such as the Janssen (Johnson & Johnson) or the AstraZeneca COVID-19 vaccines. This may be more likely to happen if the vaccines are given at short intervals. However, the immune response to the AstraZeneca COVID-19 vaccine was not affected in a study of individuals who received a previous ChAdOx1-vectored vaccine for a non-spike transgene at least one year earlier(68, 69).

Other studies suggest that an interval of three months between administrations of two adenoviral vectored vaccines reduces the risk of this interference(70-74). For this reason, we will enrol participants who have been vaccinated with an mRNA Covid vaccine or will have their NHS Covid vaccine offer at least three months after the ChAdOx1 RVF vaccine. No such theoretical interference will be expected with mRNA or protein COVID-19 vaccines (such as Moderna, Pfizer, Novavax) and these can be given at least 2 weeks before or after the ChAdOx1 RVF.

5.4 Known Potential Benefits

Volunteers will not benefit directly from participation in this study. However, it is hoped that the information gained from this study will contribute to the development of a safe and effective RVFV vaccine regimen. The only benefits for participants would be information about their general health status.

©Oxford University

6. RECRUITMENT AND WITHDRAWAL OF TRIAL VOLUNTEERS

6.1 Volunteers

Volunteers may be recruited by use of an advertisement +/- registration form formally approved by the ethics committee(s) and distributed or posted in the following places:

- In public places, including buses and trains, with the agreement of the owner/proprietor.
- In newspapers or other literature for circulation.
- On radio via announcements.
- On a website or social media site operated by our group or with the agreement of the owner or operator (including on-line recruitment through our web-site).
- By e-mail distribution to a group or list only with the express agreement of the network administrator or with equivalent authorisation.
- By email distribution to individuals who have already expressed an interest in taking part in any clinical trial at the Oxford Vaccine Centre.
- On stalls or stands at exhibitions or fairs.
- Via presentations (e.g. presentations at lectures or invited seminars).
- Direct mail-out: This will involve obtaining names and addresses of adults via the most recent Electoral Roll. The contact details of individuals who have indicated that they do not wish to receive postal mail-shots would be removed prior to the investigators being given this information. The company providing this service is registered under the relevant data protection legislation. Investigators would not be given dates of birth or ages of individuals but the list supplied would only contain names of those aged between 18-50 years (as per the inclusion criteria).
- Oxford Vaccine Centre databases: We may contact individuals from databases of groups within the CCVTM (including the Oxford Vaccine Centre database) of previous trial participants who have expressed an interest in receiving information about all future studies for which they may be eligible.

6.2 Informed consent

All volunteers will sign and date the informed consent form before any study specific procedures are performed. The information sheet will be made available to the volunteer at least 24 hours prior to the screening visit. At the screening visit, the volunteer will be fully informed of all aspects of the trial, the potential risks and their obligations. The following general principles will be emphasised:

- Participation in the study is entirely voluntary
- Refusal to participate involves no penalty or loss of medical benefits
- The volunteer may withdraw from the study at any time
- The volunteer is free to ask questions at any time to allow him or her to understand the purpose of the study and the procedures involved

©Oxford University

- The study involves research of an investigational vaccine
- There is no direct benefit from participating
- The volunteer's GP will be contacted to corroborate their medical history. Volunteers will only be enrolled in the study if written or verbal information regarding the volunteer's medical history is obtained from the GP. This can either be via the study team accessing patient's electronic care summaries from local systems or by contacting the GP.
- The volunteer's blood samples taken as part of the study will be stored indefinitely and samples may be sent outside of the UK and Europe to laboratories in collaboration with the University of Oxford. These will be anonymised.

The aims of the study and all tests to be carried out will be explained. The volunteer will be given the opportunity to ask about details of the trial, and will then have time to consider whether or not to participate. If they do decide to participate, they will sign and date one consent form from which copies are made , one for them to take away and keep, and one to be stored in the case report form (CRF) – this is a paper or electronic document used to collect data relating to a particular volunteer. These forms will also be signed and dated by the Investigator.

Whilst the COVID-19 pandemic continues to affect the UK volunteers will be provided with an information sheet detailing the precautions being taken. They will be asked to agree to abide by the most recent UK government guidance on self-isolation, testing and social distancing.

6.3 Inclusion and exclusion criteria

This study will be conducted in healthy adults, who meet the following inclusion and exclusion criteria:

6.3.1 Inclusion Criteria

The volunteer must satisfy all the following criteria to be eligible for the study:

1. Healthy adults aged 18 to 50 years
2. Able and willing (in the Investigator's opinion) to comply with all study requirements
3. Willing to allow the investigators to discuss the volunteer's medical history with their General Practitioner or access this medical history electronically
4. For females only, willingness to practice continuous effective contraception (see below) for at least 3 months from the final vaccination procedure and a negative pregnancy test on the day(s) of screening and vaccination
5. Agreement to refrain from blood donation during the course of the study
6. Able to provide written informed consent

©Oxford University

6.3.2 Exclusion Criteria

The volunteer may not enter the study if any of the following apply:

1. Participation in another research study involving receipt of an investigational product in the 30 days preceding enrolment, or planned use during the study period
2. Prior receipt of an investigational vaccine likely to impact on interpretation of the trial data.
3. Prior receipt of any vaccines administered ≤30 days before enrolment and/or planned receipt of a vaccine ≤30 days after enrolment EXCEPT for protein, RNA (or other non-adenovirus based) COVID-19 vaccinations which may be given within 14 days of the trial vaccine.
4. Receipt of a recombinant simian adenoviral vaccine prior to enrolment
5. Planned receipt of another adenoviral vectored vaccine (e.g. Oxford/Astrazeneca or Janssen COVID-19 vaccines) within 90 days after the vaccination with the ChAdOx1 RVF
6. Administration of immunoglobulins and/or any blood products within the three months preceding the planned administration of the vaccine candidate
7. Any confirmed or suspected immunosuppressive or immunodeficient state, including HIV infection; asplenia; recurrent, severe infections and chronic (more than 14 days) immunosuppressant medication within the past 6 months (inhaled and topical steroids are allowed)
8. COVID-19 infection diagnosed in the community in the 28 days prior to enrolment
9. Any main covid-19 symptom within 28 days of enrolment:
    a. Fever (subjective or ≥37.8)
    b. New continuous cough
    c. Loss of sense of smell
    d. Loss of sense of taste
10. Clinical suspicion of acute COVID-19 in the 28 days prior to enrolment
11. History of allergic disease or reactions likely to be exacerbated by any component of the vaccine
12. Any history of hereditary angioedema, acquired angioedema, or idiopathic angioedema.
13. Any history of anaphylaxis in relation to vaccination
14. Pregnancy, lactation or willingness/intention to become pregnant during the study
15. History of cancer (except basal cell carcinoma of the skin and cervical carcinoma in situ)
16. History of serious psychiatric condition likely to affect participation in the study
17. Bleeding disorder (eg. factor deficiency, coagulopathy or platelet disorder), or prior history of significant bleeding or bruising following IM injections or venepuncture
18. Any other serious chronic illness requiring hospital specialist supervision
19. Suspected or known current alcohol abuse as defined by an alcohol intake of greater than 42 units every week

©Oxford University

20. Suspected or known injecting drug abuse in the 5 years preceding enrolment
21. Seropositive for hepatitis B surface antigen (HBsAg)
22. Seropositive for hepatitis C virus (antibodies to HCV)
23. Any clinically significant abnormal finding on screening biochemistry or haematology blood tests or urinalysis
24. Any other significant disease, disorder or finding which may significantly increase the risk to the volunteer because of participation in the study, affect the ability of the volunteer to participate in the study or impair interpretation of the study data
25. Inability of the study team to contact the volunteer's GP (or access summary care record, if available) to confirm medical history and safety to participate
26. Prior natural exposure to RVFV as determined by seropositivity for RVFV antigens by ELISA and neutralizing antibody assay (serology will be requested at the discretion of the investigator)
27. History of confirmed major thrombotic event (including cerebral venous sinus thrombosis, deep vein thrombosis, pulmonary embolism), history of antiphospholipid syndrome, or history of heparin induced thrombocytopenia

6.3.3 Effective contraception for female volunteers of childbearing potential

Female volunteers are required to use an effective form of contraception during the course of the study, for at least 3 months after their final vaccination procedure. As this is a Phase I, `first-in-human, study there is no information about the effect of this vaccine on a foetus. Male subjects with female partners of child-bearing potential are not required to use barrier methods for the purposes of contraception whilst taking part in this study as the risk of excretion of the vaccine is negligible.

Acceptable forms of contraception for female volunteers include:

- Established use of oral, injected or implanted hormonal methods of contraception.
- Placement of an intrauterine device (IUD) or intrauterine system (IUS).
- Barrier methods of contraception (condom or occlusive cap with spermicide)
- Male sterilisation, if the vasectomised partner is the sole partner for the subject.
- True abstinence: when this is in line with the preferred and usual lifestyle of the subject. Periodic abstinence (e.g. calendar, ovulation, symptothermal, post-ovulation methods), declaration of abstinence for the duration of exposure to IMP, and withdrawal are not acceptable methods of contraception

Under this protocol, a woman is considered of childbearing potential (WOCBP), i.e. fertile, following menarche and until becoming post-menopausal unless permanently sterile. Permanent sterilisation methods include hysterectomy, bilateral salpingectomy and

©Oxford University

bilateral oophorectomy. A postmenopausal state is defined as no menses for 12 months without an alternative medical cause.

6.3.4 Prevention of 'Over Volunteering'

Volunteers will be excluded from the study if they are concurrently involved in another trial. In order to check this, volunteers will be asked to provide their National Insurance or Passport number (if they are not entitled to a NI number) and will be registered on a national database of participants in clinical trials (*www.tops.org.uk*).

6.3.5 Criteria for postponement of vaccination

The following events constitute contraindications to administration of the vaccine at that point in time; if any one of these events occurs at the time scheduled for vaccination, the subject may instead be vaccinated at a later date following resolution and within the window of screening procedures (defined in section 7.4.1), or withdrawn at the discretion of the Investigator.

- Acute disease at the time of vaccination. Acute disease is defined as the presence of a moderate or severe illness with or without fever.
- Temperature of >37.5°C (99.5°F) at the time of vaccination.

6.3.6 Withdrawal of Volunteers

In accordance with the principles of the current revision of the Declaration of Helsinki and any other applicable regulations, a volunteer has the right to withdraw from the study at any time and for any reason, and is not obliged to give his or her reasons for doing so. The Investigator may withdraw the volunteer at any time in the interests of the volunteer's health and well-being. In addition the volunteer may withdraw/be withdrawn for any of the following reasons:

- Administrative decision by the Investigator.
- Ineligibility (either arising during the study or retrospectively, having been overlooked at screening).
- Significant protocol deviation.
- Volunteer non-compliance with study requirements.
- An AE, which requires discontinuation of the study involvement or results in inability to continue to comply with study procedures.

The reason for withdrawal will be recorded in the CRF. If withdrawal is due to an AE, appropriate follow-up visits or medical care will be arranged, with the agreement of the volunteer, until the AE has resolved, stabilised or a non-trial related causality has been assigned. Any volunteer who is withdrawn from the study may be replaced, if that is possible

©Oxford University

within the specified time frame. The Local Safety Monitor (LSM) may recommend withdrawal of volunteers.

Any volunteer who fails to attend for two or more follow-up visits during the study will be deemed to have withdrawn from the study.

If a volunteer withdraws from the study, blood samples collected before their withdrawal from the trial will be used/ stored unless the volunteer specifically requests otherwise.

In all cases of subject withdrawal, excepting those of complete consent withdrawal, long-term safety data collection, including some procedures such as safety bloods, will continue as appropriate if subjects have received one or more vaccine doses.

6.4 Compliance with Dosing Regime

All doses in this vaccine study will be administered by the Investigator and recorded in the CRF. The study medication will be at no time in the possession of the volunteer and compliance will, therefore, not be an issue.

6.5 Pregnancy

Should a volunteer become pregnant during the trial, she will be followed up as other volunteers and in addition will be followed until pregnancy outcome. We will not routinely perform venepuncture in a pregnant volunteer.

©Oxford University

7. CLINICAL PROCEDURES

This section describes the clinical procedures for evaluating study participants and follow-up after administration of study vaccine.

7.1 Study procedures

All volunteers will have the same schedule of clinic attendances and procedures as indicated in the schedules of attendance (Table 5). All subjects will receive the ChAdOx1 RVF vaccine, and undergo follow-up for a total of 12 weeks. The total volume of blood donated during the study will be 300mL. Additional visits or procedures may be performed at the discretion of the investigators, e.g., further medical history and physical examination, urine microscopy in the event of positive urinalysis or additional blood tests if clinically relevant.

7.2 Observations

Pulse, blood pressure and temperature will be measured at the time-points indicated in the schedule of procedures and may also be measured as part of a physical examination if indicated at other time-points.

7.3 Blood Tests and Urinalysis

Blood will be drawn for the following laboratory tests and processed:

1. At Oxford University Hospitals' NHS Trust using NHS standard procedures:

- **Haematology;** Full Blood Count
- **Biochemistry;** Sodium, Potassium, Urea, Creatinine, Albumin, Liver Function Tests (ALT, ALP, Bilirubin)
- **Diagnostic serology;** HBsAg, HCV antibodies, HIV antibodies (specific consent will be gained prior to testing blood for these blood-borne viruses)
- **Immunology;** Human Leukocyte Antigen (HLA) typing

Additional safety blood tests may be performed if clinically relevant at the discretion of the medically qualified investigators. These generally include, but are not limited to AST, GGT and a coagulation screen.

2. At University of Oxford research laboratories:

- **Exploratory Immunology;** Immunogenicity will be assessed by a variety of immunological assays. These may include antibodies to Gn and Gc proteins by ELISA and virus neutralising assays, ex vivo ELISpot assays for interferon gamma and flow cytometry assays, functional antibody assays and B cell analyses. Other exploratory immunological assays including cytokine analysis and other antibody assays, DNA

©Oxford University

analysis of genetic polymorphisms potentially relevant to vaccine immunogenicity and gene expression studies amongst others may be performed at the discretion of the Investigators.

3. **Urinalysis;** Urine will be tested for protein, blood and glucose at screening. For female volunteers only, urine will be tested for beta-human chorionic gonadotrophin (β-HCG) at screening and immediately prior to each vaccination.

Collaboration with other specialist laboratories in the UK, Europe and outside of Europe for further exploratory immunological tests may occur. This would involve the transfer of serum or plasma and/or PBMC to these laboratories, but these would remain anonymised. Volunteers will be asked for their consent for this before enrollment. Immunological assays will be conducted according to local SOPs.

Subjects will be informed that there may be leftover samples of their blood (after all testing for this study is completed), and that such samples may be stored indefinitely in the Oxford Vaccine Center Biobank for possible future research (exploratory immunology), including human DNA and RNA analyses to search for correlates of vaccine immunogenicity and efficacy. Subjects will be able to decide if they will permit such future use of any leftover samples. If a subject elects not to permit this, all of that subject's leftover samples will be discarded after the required period of storage to meet Good Clinical Practice (GCP) and regulatory requirements. Urine samples will be destroyed after analysis and will not be stored for future research use.

7.4 Study visits

The study visits and procedures will be undertaken by one of the clinical trials team. The procedures to be included in each visit are documented in the schedule of attendances (Table 5). Each visit is assigned a time-point and a window period, within which the visit will be conducted.

7.4.1 Screening visit

All potential volunteers will have a screening visit, which may take place up to 90 days prior to vaccination. Informed consent will be taken before screening, as described in section 6.2. If consent is obtained, the screening procedures indicated in the schedule of attendances will be undertaken. To avoid unnecessary additional venepuncture, if the appropriate blood test results for screening are available for the same volunteer from a screening visit for another Jenner Institute Clinical Trials group vaccine study, these results may be used for assessing eligibility (provided the results date is within the 3 months preceding enrolment in RVF001).

©Oxford University

The subject's general practitioner will be contacted with the written permission of the subject after satisfactory screening as notification that the subject has volunteered for the study and to ascertain any significant medical history. During the screening the volunteers will be asked to provide their National Insurance or passport number so that this can be entered on to a national database which helps prevent volunteers from participating in more than one clinical trial simultaneously or over-volunteering for clinical trials (www.tops.org.uk).

Abnormal clinical findings from the urinalysis or blood tests at screening will be assessed by the lead clinician according to the relevant SOP. Abnormal blood tests following screening will be assessed according to site-specific laboratory adverse event grading tables which are filed in the trial master file (TMF) or the Investigator Site File (ISF). Any abnormal test result deemed clinically significant may be repeated to ensure it is not a single occurrence. If an abnormal finding is deemed to be clinically significant, the volunteer will be informed and appropriate medical care arranged with the permission of the volunteer.

The eligibility of the volunteer will be reviewed by the study clinician at the end of the screening visit and again when all results from the screening visit have been considered. Decisions to exclude the volunteer from enrolling in the trial or to withdraw a volunteer from the trial will be at the discretion of the Investigator. If eligible, a day 0 visit will be scheduled for the volunteer to receive the vaccine.

7.4.2 Day 0: Enrolment and Vaccination Visit

Volunteers will not be considered enrolled in the study until they have received a vaccine. Before vaccination, the eligibility of the volunteer will be reviewed including re-checking informed consent and willingness to be vaccinated. Pulse, blood pressure and temperature will be observed and if necessary, a medical history and physical examination may be undertaken to determine need to postpone vaccination depending on criteria listed in section 6.3.5. Vaccinations will be administered as described below.

7.4.2.1 Vaccinations

Before each vaccination, the on-going eligibility of the volunteer will be reviewed. All vaccines will be administered intramuscularly according to SOP VC002 Vaccination as described below in section 8.4 and the vaccine dilution SOP when required. The injection site will be covered with a sterile dressing and the volunteer will stay in the CCVTM for observation, in case of immediate adverse events. Observations will be taken 30 minutes after vaccination (+/- 5 minutes) and the sterile dressing removed and injection site inspected. Observations will also be taken at 60 minutes (+/- 10 minutes) before the volunteer leaves. An oral thermometer, tape measure and diary card (paper or electronic) will be given to each volunteer, with instructions on use, along with the emergency 24 hour telephone number to contact the on-call study physician if needed.

Diary cards will collect information on the timing and severity of the following solicited AEs:

©Oxford University

Table 3. Solicited AEs as collected on post vaccination diary cards

Local solicited AEs	Systemic solicited AEs
Pain	Fever
Redness	Feverishness
Warmth	Joint pains
Itch	Muscle pains
	Fatigue
	Headache
	Nausea
	Malaise

Volunteers will be instructed on how to self-assess the severity of these AEs. There will also be space on the diary card to self-document unsolicited AEs, and whether medication was taken to relieve the symptoms.

7.4.2.2 Sequence of Enrolment and Vaccination of Volunteers

For safety reasons, the first volunteer in Group 1 will be vaccinated ahead of any other volunteers and the profile of adverse events will be reviewed after 48 hours (±24h) post vaccination. Provided there are no safety concerns, as assessed by the CI and the Local Safety Monitor (LSM), another 2 volunteers will be vaccinated at the same dose after at least 48 hours has elapsed following the first volunteer being vaccinated and at least 1 hour apart from each other. An independent safety review will be conducted by the LSM after vaccination of all 3 volunteers in group 1. This review will include an assessment of the profile of adverse events and the results of safety blood tests at day 7 post vaccination. The CI and the LSM will be asked to provide the decision on whether to proceed with vaccinations of the first volunteer to receive the next incremental dose in group 2. If there are no safety concerns, the first volunteer in group 2 may be vaccinated.

Enrolment of the first volunteer in Group 2 will only proceed if the CI and LSM assess the data from the first three vaccinees in Group 1 as indicating that it is safe to do so. The first subject in Group 2 will be vaccinated alone, and a 48 hour gap allowed before vaccinating further subjects in this group. Provided there are no safety concerns, as assessed by the CI and the LSM, another 2 volunteers will be vaccinated at the same dose after at least 48 hours has elapsed following the first volunteer being vaccinated and at least 1 hour apart from each other and will be reviewed in a further 48h (±24h). Provided there are no safety concerns as assessed by the CI and LSM, the remaining participants in the group may be vaccinated. An

©Oxford University

independent safety review will be conducted by the LSM after vaccination of all 6 volunteers. This review will include an assessment of the profile of adverse events and the results of safety blood tests at day 7 post vaccination. The CI and the LSM will be asked to provide the decision on whether to proceed with vaccinations of the first volunteer to receive the next incremental dose in group 3. If there are no safety concerns, the first volunteer in group 3 may be vaccinated.

Enrolment of the first volunteer in Group 3 will only proceed if the CI and LSM assess the data from all 6 vaccinees in Group 2 as indicating that it is safe to do so. The first subject in Group 3 will be vaccinated alone, and a 48 hour gap allowed before vaccinating further subjects in this group. Provided there are no safety concerns, as assessed by the CI and the LSM, another 2 volunteers will be vaccinated at the same dose after at least 48 hours has elapsed following the first volunteer being vaccinated and at least 1 hour apart from each other and will be reviewed in a further 48h (±24h). Provided there are no safety concerns as assessed by the CI and LSM, the remaining participants in the group may be vaccinated.

The enrolment sequence is summarised below (table 4):

Table 4. Sequence of enrolment

Sequence	Volunteer groups	Minimum Interval before next step	Local Safety Monitor Interim Safety Review
Step 1	Group 1 first volunteer	48 hours	Y
Step 2	Group 1 second volunteer	1 hour	N
Step 3	Group 1 third volunteer	7 days	Y
Step 4	Group 2 first volunteer	48 hours	Y
Step 5	Group 2 second volunteer	1 hour	N
Step 6	Group 2 third volunteer	48 hours	Y
Step 7	Group 2 remaining volunteers	7 days	Y
Step 8	Group 3 first volunteer	48 hours	Y
Step 9	Group 3 second volunteer	1 hour	N
Step 10	Group 3 third volunteer	48 hours	Y
Step 11	Group 3 remaining volunteers	n/a	n/a

7.4.3 Subsequent visits: days 2, 7, 14, 28, 56 and 84

Follow-up visits will take place 48 hours (±24h), 7 days (± 2 days), 14 days (± 3 days), 28 days (± 3 days), 56 days (± 7 days) and 84 days (± 7 days) . Volunteers will be assessed for local and systemic adverse events, interim history, physical examination, review of diary cards (paper or electronic) and blood tests at these time points as detailed in the schedule of attendances. Blood will also be taken for exploratory immunology purposes. Photographs of the injection site may be taken with the volunteer's consent and at the investigator's discretion.

If volunteers experience adverse events (laboratory or clinical), which the investigator (physician), CI and/or LSM determine necessary for further close observation, the volunteer may be admitted to an NHS hospital for observation and further medical management under the care of the Consultant on call.

©Oxford University

At the end of the study, GP's will be notified about the status of their patient's enrolment in the trial.

7.4.4 COVID-19 Study Considerations

The study will be carried out in line with the latest Public Health England (PHE) advice. Local SOPs for the use of appropriate personal protective equipment (PPE) and social distancing will be followed by clinical staff.

Participants must consent to inform the trial team of covid-19 infection during the study as part of the informed consent process, in order to be enrolled in the study. Participants will be reminded to follow the up to date local public health guidance regarding COVID-19 during the trial. Inkeeping with PHE guidance on vaccine associated fever during the pandemic (75), participants experiencing fever within the first 48 hours of vaccination, with no other primary symptom of covid-19 (cough, anosmia, ageusia) will not be required to self-isolate unless there is otherwise clinical suspicion of COVID-19.

Participants will be able to discuss symptoms with a medically qualified oncall study clinician via mobile phone at all times during the study. Participants describing potential COVID-19 symptoms may be recommend to seek follow up and testing for COVID-19 through healthcare pathways available to them.

©Oxford University

Table 5. Schedule of attendances

Attendance Number	1[S]	2	3	4	5	6	7	8
Timeline** (days)	≤ 90	0	2	7	14	28	56	84
Time window (days)			±1	±2	±3	±3	±7	±7
Informed Consent	X							
Review contraindications, inclusion and exclusion criteria	X	X						
Vaccination		X						
Vital signs^	X	X	X	X	X	X	X	X
Ascertainment of adverse events		X	X	X	X	X	X	X
Diary cards provided		X						
Diary cards collected						X		
Medical History, Physical Examination	X	(X)	(X)	(X)	(X)	(X)	(X)	(X)
Biochemistry[$], Haematology (mL)	5	5	5	5		5		
Exploratory immunology[£] (mL)		50		10	50	50	50	50
Urinalysis	X							
Urinary β–HCG (women only)	X	X						
HLA typing (mL)		4						
HBsAg, HCV Ab, HIV serology (mL)	5							
Blood volume per visit	10	59	5	15	50	55	50	50
Cumulative blood volume[%]	10	69	74	89	139	194	244	294

S = screening visit; (X) = if considered necessary ^ = Vital signs includes pulse, blood pressure and temperature; $ = Biochemistry will include Sodium, Potassium, Urea, Creatinine, Albumin and Liver function tests. £ = Exploratory immunology includes antibodies to RVFV, ex vivo interferon-gamma ELISpot responses to RVFV

** Timeline is approximate only. Exact timings of visits relate to the day on enrolment, ie, each visit must occur at indicated number of days after enrolment ± time window.

% Cumulative blood volume for Oxford volunteers if blood taken as per schedule, and excluding any repeat safety blood test that may be necessary.

©Oxford University

8. INVESTIGATIONAL PRODUCTS

The following vaccinations will be given in this study:

1. ChAdOx1 RVF 5 x 10^9vp
2. ChAdOx1 RVF 2.5 x 10^{10}vp
3. ChAdOx1 RVF 5 x 10^{10}vp

8.1. Manufacturing and Presentation

8.1.1 Description of ChAdOx1 RVF

ChAdOx1 RVF vaccine consists of the replication-deficient simian adenovirus vector ChAdOx1, encoding the RVFV envelope glycoproteins (Gn and Gc) under the control of the human CMV major immediate early promoter.

8.1.2 ChAdOx1 RVF formulation and packaging

ChAdOx1 RVF is manufactured by Advent S.r.l., Pomezia, Italy, in formulation buffer at a nominal concentration of >1.0 x 10^{11} vp/mL . The drug product is filled into 3 mL glass vials (supplied sterile by Nuova Ompi) with a 13 mm grey chlorobutyl rubber freeze-dry injection stopper (supplied sterile by West Pharma) and a 13 mm aluminium Flip Off seal (supplied sterile by West Pharma). The vials are supplied sterile. The containers and closures are tested for compliance with defined specifications. The vials are made from Ph Eur Type 1 glass.

8.2 Supply

ChAdOx1 RVF has been formulated and vialed under Good Manufacturing Practice conditions at ADVENT S.R.L., Italy. At the Clinical Biomanufacturing Facility (CBF), University of Oxford, the vaccine will be certified and labelled for the trial by a Qualified Person (QP) before transfer to the clinical site.

8.3 Storage

The vaccine is stored at nominal -80°C in a locked freezer, at the clinical site. All movements of the study vaccines will be documented in accordance with existing standard operating procedure (SOP). Vaccine accountability, storage, shipment and handling will be in accordance with relevant SOPs and forms.

8.4 Administration of Investigational Medicinal Products

On vaccination day, ChAdOx1 RVF will be allowed to thaw to room temperature and will be administered within 1 hour of removal from the freezer. The vaccine will be administered intramuscularly into the deltoid of the non-dominant arm (preferably). All volunteers will be observed in the unit for 1 hour (±10 minutes) after vaccination. During administration of the

©Oxford University

investigational products, Advanced Life Support drugs and resuscitation equipment will be immediately available for the management of anaphylaxis. Vaccination will be performed and the IMPs handled according to the relevant SOPs.

8.5 Minimising environmental contamination with genetically modified organisms (GMO)

The study will be performed in accordance with UK Genetically Modified Organisms (Contained Use) Regulations (2014). In order to minimise dissemination of the recombinant vectored vaccine virus into the environment, inoculation sites will be covered with a dressing after immunisation. This should absorb any virus that may leak out through the needle track. The dressing will be removed from the injection site after 30 minutes (+15/- 5 minutes) and will be disposed as GMO waste by autoclaving.

©Oxford University

9. ASSESSMENT OF SAFETY

Safety will be assessed by the frequency, incidence and nature of adverse events and serious adverse events arising during the study.

9.1 Definitions

9.1.1 Adverse Event (AE)

An AE is any untoward medical occurrence in a volunteer, which may occur during or after administration of an Investigational Medicinal Product (IMP) and does not necessarily have a causal relationship with the intervention. An AE can therefore be any unfavourable and unintended sign (including an abnormal laboratory finding), symptom or disease temporally associated with the study intervention, whether or not considered related to the study intervention.

9.1.2 Adverse Reaction (AR)

An AR is any untoward or unintended response to an IMP. This means that a causal relationship between the IMP and an AE is at least a reasonable possibility, i.e., the relationship cannot be ruled out. All cases judged by the reporting medical Investigator as having a reasonable suspected causal relationship to an IMP (i.e. possibly, probably or definitely related to an IMP) will qualify as adverse reactions.

9.1.3 Unexpected Adverse Reaction

An adverse reaction, the nature or severity of which is not consistent with the applicable product information (e.g., IB for an unapproved IMP).

9.1.4 Serious Adverse Event (SAE)

An SAE is an AE that results in any of the following outcomes, whether or not considered related to the study intervention.

- Death
- Life-threatening event (i.e., the volunteer was, in the view of the Investigator, at immediate risk of death from the event that occurred). This does not include an AE that, if it occurred in a more severe form, might have caused death.
- Persistent or significant disability or incapacity (i.e., substantial disruption of one's ability to carry out normal life functions).
- Hospitalisation, regardless of length of stay, even if it is a precautionary measure for continued observation. Hospitalisation (including inpatient or outpatient hospitalisation for an elective procedure) for a pre-existing condition that has not worsened unexpectedly does not constitute a serious AE.
- An important medical event (that may not cause death, be life threatening, or require hospitalisation) that may, based upon appropriate medical judgment, jeopardise the

©Oxford University

volunteer and/or require medical or surgical intervention to prevent one of the outcomes listed above. Examples of such medical events include allergic reaction requiring intensive treatment in an emergency room or clinic, blood dyscrasias, or convulsions that do not result in inpatient hospitalisation.

- Congenital anomaly or birth defect.

Hospitalisations for elective procedures that are planned prior to enrolment into the study will not be reported as SAEs.

9.1.5 Serious Adverse Reaction (SAR)

An adverse event (expected or unexpected) that is both serious and, in the opinion of the reporting Investigator or Sponsors, believed to be possibly, probably or definitely due to an IMP or any other study treatments, based on the information provided.

9.1.6 Suspected Unexpected Serious Adverse Reaction (SUSAR)

A serious adverse reaction, the nature and severity of which is not consistent with the information about the medicinal product in question set out in the IB or Summary of Product Characteristics (SmPC).

9.2 Foreseeable Adverse Reactions:

The foreseeable ARs following vaccination with ChAdOx1 RVF include injection site pain, erythema, warmth, swelling, pruritus, myalgia, arthralgia, headache, fatigue, fever, feverishness, malaise and nausea.

9.3 Expected Serious Adverse Events

No serious adverse events are expected in this study.

9.4 Causality Assessment

For every AE, an assessment of the relationship of the event to the administration of the vaccine will be undertaken by the CI-delegated clinician. An intervention-related AE refers to an AE for which there is a possible, probable or definite relationship to administration of a vaccine. An interpretation of the causal relationship of the intervention to the AE in question will be made, based on the type of event; the relationship of the event to the time of vaccine administration; and the known biology of the vaccine therapy (Table 6). Alternative causes of the AE, such as the natural history of pre-existing medical conditions, concomitant therapy, other risk factors and the temporal relationship of the event to vaccination will be considered and investigated. Causality assessment will take place during planned safety reviews, interim

©Oxford University

analyses (e.g. if a holding or stopping rule is activated) and at the final safety analysis, except for SAEs, which should be assigned by the reporting investigator.

Table 6. Guidelines for assessing the relationship of vaccine administration to an AE.

0	**No Relationship**	No temporal relationship to study product ***and***<br>Alternate aetiology (clinical state, environmental or other interventions); ***and***<br>Does not follow known pattern of response to study product
1	**Unlikely**	Unlikely temporal relationship to study product ***and***<br>Alternate aetiology likely (clinical state, environmental or other interventions) ***and***<br>Does not follow known typical or plausible pattern of response to study product.
2	**Possible**	Reasonable temporal relationship to study product; ***or***<br>Event not readily produced by clinical state, environmental or other interventions; ***or***<br>Similar pattern of response to that seen with other vaccines
3	**Probable**	Reasonable temporal relationship to study product; ***and***<br>Event not readily produced by clinical state, environment, or other interventions ***or***<br>Known pattern of response seen with other vaccines
4	**Definite**	Reasonable temporal relationship to study product; ***and***<br>Event not readily produced by clinical state, environment, or other interventions; ***and***<br>Known pattern of response seen with other vaccines

9.5 Reporting Procedures for All Adverse Events (see SOP VC027)

All local and systemic AEs occurring in the 28 days following each vaccination observed by the Investigator or reported by the volunteer, whether or not attributed to study medication, will be recorded (excluding those expected consequences from venepuncture, described in section 5.3). Recording and reporting of all AEs will take place as detailed in SOP VC027. All AEs that result in a volunteer's withdrawal from the study will be followed up until a satisfactory resolution occurs, or until a non-study related causality is assigned (if the volunteer consents to this). Serious adverse events (SAEs) will be collected throughout the entire trial period.

©Oxford University

9.5.1 Reporting Procedures for Serious AEs (see SOP OVC005 Safety Reporting)

In order to comply with current regulations on serious adverse event reporting to regulatory authorities, the event will be documented accurately and notification deadlines respected. SAEs will be reported on the SAE forms to members of the study team immediately the Investigators become aware of their occurrence, as described in SOP OVC005. Copies of all reports will be forwarded for review to the Chief Investigator (as the Sponsor's representative) within 24 hours of the Investigator being aware of the suspected SAE. The local safety monitor (LSM) will be notified of SAEs that are deemed possibly, probably or definitely related to study interventions; the LSM will be notified immediately (within 24 hours) of the Investigators' being aware of their occurrence. SAEs will not normally be reported immediately to the ethical committee(s) unless there is a clinically important increase in occurrence rate, an unexpected outcome, or a new event that is likely to affect safety of trial volunteers, at the discretion of the Chief Investigator and/or LSM. In addition to the expedited reporting above, the Chief Investigator (on behalf) of the Sponsor shall include all SAEs in the annual Development Safety Update Report (DSUR) report.

Hospitalisations for elective procedures that are planned prior to enrolment into the study will not be reported as SAEs.

9.5.2 Reporting Procedures for SUSARS

The Chief Investigator will report all SUSARs to the MHRA and ethical committee(s) within required timelines (15 days for all SUSARs, unless life threatening in which case 7 days, with a final report within a further 8 days (total 15). The Chief Investigator will also inform all Investigators concerned of relevant information about SUSARs that could adversely affect the safety of participants. All SUSARs and deaths occurring during the study will be reported to the Sponsor. For all deaths, available autopsy reports and relevant medical reports will be made available for reporting to the relevant authorities.

9.5.3 Development Safety Update Report

A Development Safety Update Report (DSUR) will be submitted by the CI to the competent authority and ethical committee on the anniversary of the first approval date from the regulatory authority for each IMP.

9.6 Assessment of severity

The severity of clinical and laboratory adverse events will be assessed according to the scales in Tables 6-8, also described in the SOP VC027.

©Oxford University

Table 7. Severity grading criteria for local adverse events.

Adverse Event	Grade	Intensity
Erythema at injection site*	1	>3 - ≤50 mm
	2	>50 - ≤100 mm
	3	>100 mm
Swelling at injection site	1	>3 - ≤20 mm
	2	>20 - ≤50 mm
	3	>50 mm
Ulceration/necrosis of skin at injection site	1	-
	2	-
	3	Any

*erythema or swelling ≤3mm is an expected consequence of skin puncture and will therefore not be considered an adverse event.

Table 8. Severity grading criteria for physical observations

	Grade 1 (mild)	Grade 2 (moderate)	Grade 3 (severe)
Fever (oral)	37.6°C - 38.0°C	38.1°C – 39.0°C	>39.0°C
Tachycardia (bpm)*	101 - 115	116 – 130	>130
Bradycardia (bpm)**	50 – 54	40 – 49	<40
Systolic hypertension (mmHg)	141 - 159	160 – 179	≥180
Systolic hypotension (mmHg)***	85 - 89	80 – 84	<80
Diastolic hypertension (mmHg)	91 - 99	100 – 109	≥110

*Taken after ≥10 minutes at rest
**Use clinical judgement when characterising bradycardia among some healthy subject populations, for example, conditioned athletes.
***Only if symptomatic (e.g. dizzy/ light-headed)

©Oxford University

Table 9. Severity grading criteria for local and systemic AEs.

**GRADE 0**	None: Symptom not experienced
**GRADE 1**	Mild: Short-lived or mild symptoms; medication may be required. No limitation to usual activity
**GRADE 2**	Moderate: Mild to moderate limitation in usual activity. Medication may be required.
**GRADE 3**	Severe: Considerable limitation in activity. Medication or medical attention required.

9.7 Procedures to be followed in the event of abnormal findings

Laboratory parameters for inclusion/exclusion in the trial will be considered on an individual basis, with investigator discretion for interpretation of results and the need for repeated tests. Laboratory adverse events will be assessed using the tables as detailed in SOP VC027. Abnormal clinical findings from medical history, examination or blood tests will be assessed as to their clinical significance throughout the trial. If a test is deemed clinically significant, it may be repeated, to ensure it is not a single occurrence. If a test remains clinically significant, the volunteer will be informed and appropriate medical care arranged as appropriate and with the permission of the volunteer. Decisions to exclude the volunteer from enrolling in the trial or to withdraw a volunteer from the trial will be at the discretion of the Investigator.

9.8 Local Safety Monitor

A Local Safety Monitor (LSM) will be appointed to provide real-time safety oversight. The LSM will review SAEs deemed possibly, probably or definitely related to study interventions. The LSM will be notified within 24 hours of the Investigators' being aware of their occurrence. The LSM has the power to place the study on hold if deemed necessary following a study intervention-related SAE. At the time of writing the LSM will be Dr Brian Angus, a Clinical Tutor in Medicine, Honorary Consultant Physician and Director, Centre for Tropical Medicine at the University of Oxford.. All correspondence between Investigator and LSM will be conveyed by the Investigator to the trial Sponsor.

The LSM may be contacted for advice and independent review by the Investigator or trial Sponsor in the following situations:

- Following any SAE deemed to be possibly, probably, or definitely related to a study intervention.
- Any other situation where the Investigator or trial Sponsor feels independent advice or review is important.

©Oxford University

9.8.1 Interim Safety Reviews

Interim safety reviews with the LSM are schedule during the enrolment of the first volunteers in each group and prior to dose escalations, as outlined in section 7.4.2.2.

The safety profile of the IMP will be assessed on an on-going basis by the Investigators with communication to the LSM as necessary. The Chief Investigator and relevant Investigators (as per the trial delegation log) will also review safety issues and SAEs as they arise.

9.9 Safety Stopping/Holding Rules

Safety holding rules have been developed considering the fact that this is a first-in-human dose escalation study.

'Solicited adverse events' are those listed as foreseeable adverse events in section 9.2 of the protocol, occurring within the first 7 days after vaccination (day of vaccination and six subsequent days). 'Unsolicited adverse events' are adverse events other than the foreseeable AEs occurring within the first 7 days, or any AEs occurring after the first 7 days after vaccination.

9.9.1 Group holding rules

The group holding rules are described below:

- **Solicited local adverse events:**
  - If 2 or more participants experience ANY grade 3 solicited local adverse event beginning within 2 days of vaccination (day of vaccination and one subsequent day).

- **Solicited systemic adverse events:**
  - If 2 or more participants experience ANY grade 3 solicited systemic adverse event beginning within 2 days of vaccination (day of vaccination and one subsequent day).

- **Unsolicited adverse events:**
  - If 2 or more participants experience ANY grade 3 unsolicited adverse event (including laboratory adverse events) that is considered possibly, probably or definitely related to vaccination.

- **A serious adverse event considered possibly, probably or definitely related to vaccination occurs**

- **Death that is considered related to vaccination occurs**

- **A life-threatening reaction occurs**

©Oxford University

If a holding rule has been met and following an internal safety review it is deemed appropriate to restart dosing, a request to restart dosing with pertinent data must be submitted to the regulatory authority as a request for a substantial amendment. The internal safety review will consider:

- The relationship of the AE or SAE to the vaccine.
- The relationship of the AE or SAE to the vaccine dose, or other possible causes of the event.
- If appropriate, additional screening or laboratory testing for other volunteers to identify those who may develop similar symptoms and alterations to the current Participant Information Sheet (PIS) are discussed.
- New, relevant safety information from ongoing research programs on the various components of the vaccine.

The local ethics committee and vaccine manufacturers will also be notified if a holding rule is activated or released.

As per section 6.3.5, if a volunteer has an acute illness (moderate or severe illness with or without fever) or a fever (oral temperature greater than 37.5°C) at the scheduled time of administration of investigational product, the volunteer will not receive the vaccine at that time. The vaccine may be administered to that volunteer at a later date within the time window specified in the protocol (see Table 5) or they may be withdrawn from the study at the discretion of the Investigator.

All vaccinated volunteers will be followed for safety until the end of their planned participation in the study or until resolution or stabilisation (if determined to be chronic sequelae) of their AEs, providing they consent to this.

In addition to these pre-defined criteria, the study can be put on hold upon advice of the Local Safety Monitor, Chief Investigator, Study Sponsor, Regulatory Authority or Ethical Committee(s) , for any single event or combination of multiple events which, in their professional opinion, jeopardise the safety of the volunteers or the reliability of the data.

©Oxford University

10. STATISTICS

This is a descriptive safety study, where volunteers will be vaccinated with a single dose of ChAdOx1 RVF. Fifteen volunteers will be vaccinated in total. This sample size should allow an estimation to be made of the frequency and magnitude of outcome measures, rather than aiming to obtain statistical significance for differences between groups. Safety data will be presented according to frequency, severity and duration of adverse events.

The primary analysis for immunogenicity will be to assess the difference in magnitude of RVFV specific T-cell and antibody responses between the groups. We will assess vaccine immunogenicity by comparing the change in these immunological parameters from baseline to different time points.

Sample Size Selection

This is a descriptive phase I first in human trial that will balance the safety of volunteers with the aims to assess the vaccine's safety profile and immunogenicity after selected doses of the vaccines. The primary dose comparison will be between Groups 1, 2 and 3, which will have 3-6 subjects each. RVFV specific immunogenicity will be the key immunological readout assessed by a variety of immunological assays.

©Oxford University

11. DATA MANAGEMENT

11.1 Data Handling

The Chief Investigator will be responsible for all data that accrues from the study. The data will be entered into the volunteers' CRFs in a paper and/or electronic format (using OpenClinica™ database). Electronic data will be stored on secure servers which are outsourced by OpenClinica™. Data will be entered in a web browser on PCs in the CCVTM building and then transferred to the OpenClinica Database by encrypted (Https) transfer. OpenClinica™ meets FDA part 11B standards. This includes safety data, laboratory data (both clinical and immunological) and outcome data.

Adverse event data will also be entered onto electronic or paper diaries by the volunteer

11.2 Record Keeping

The Investigators will maintain appropriate medical and research records for this trial, in compliance with GCP and regulatory and institutional requirements for the protection of confidentiality of volunteers. The Chief Investigator, co-Investigators and clinical research nurses will have access to records. The Investigators will permit authorised representatives of the Sponsor(s), as well as ethical and regulatory agencies to examine (and when required by applicable law, to copy) clinical records for the purposes of quality assurance reviews, audits and evaluation of the study safety and progress.

With the volunteers' consent, we will keep their contact details after participation in the study is complete, so we may inform them of opportunities to participate in future vaccine related research. This will be entirely optional and participation in this study will not be affected by their decision to allow or not allow storage of their contact details beyond participation in this trial. Details will be stored electronically on a secure server and only authorised individuals at the CCVTM will have access to it. We will not, under any circumstances, share their contact details with any third party institutions without their permission. Volunteers will be informed that being contacted does not oblige them to agree to take part in future research and they can ask us to have their contact details removed from our database at any time.

11.3 Source Data and Case Report Forms (CRFs)

All protocol-required information will be collected in CRFs designed by the Investigators. All source documents will be filed in the CRF. Source documents are original documents, data, and records from which the volunteer's CRF data are obtained. For this study, these will include, but are not limited to, volunteer consent form, blood results, GP response letters, laboratory records, diaries, and correspondence. In the majority of cases, CRF entries will be considered source data as the CRF is the site of the original recording (i.e. there is no other written or electronic record of data). In this study this will include, but is not limited to medical

©Oxford University

history, medication records, vital signs, physical examination records, urine assessments, blood results, adverse event data and details of vaccinations. All source data and volunteer CRFs will be stored securely.

11.4 Data Protection

The study protocol, documentation, data and all other information generated will be held in strict confidence. No information concerning the study or the data will be released to any unauthorised third party, without prior written approval of the sponsor.

11.5 Data Quality

Data collection tools will undergo appropriate validation to ensure that data is collected accurately and completely. Datasets provided for analysis will be subject to quality control processes to ensure analysed data is a true reflection of the source data.

Trial data will be managed in compliance with local data management SOPs (including the overarching SOP OVC007 Data and Database Management). If additional, study specific information is required, an approved Data Management Plan will be implemented.

The trial will comply with the General Data Protection Regulation (GDPR) and Data Protection Act 2018, which requires data to be de-identified as soon as it is practical to do so.

11.6 Archiving

Study data may be stored electronically on a secure server, and paper notes will be kept in a key-locked filing cabinet at the CCVTM – Churchill Hospital, University of Oxford. All essential documents will be retained for a minimum of 5 years after the study has finished. The need to store study data for longer in relation to licensing of the vaccine will be subject to ongoing review. For effective vaccines that may be licensed, we may store research data securely at the University of Oxford for at least 15 years after the end of the study, subject to adjustments in clinical trials regulations. Participants' bank details will be stored for 7 years in line with University of Oxford financial policy.

General archiving procedures will be conducted in compliance to SOP OVC020 Archiving.

©Oxford University

12. QUALITY CONTROL AND QUALITY ASSURANCE PROCEDURES

12.1 Investigator procedures

Approved site-specific standard operating procedures (SOPs) will be used at all clinical and laboratory sites.

12.2 Monitoring

Monitoring will be performed according to the principles of GCP by Clinical Trials Research Governance (CTRG). Following written SOPs, the monitors will verify that the clinical trial is conducted and data are generated, documented and reported in compliance with the protocol, GCP and the applicable regulatory requirements. The Investigator sites will provide direct access to all trial related source data/documents and reports for the purpose of monitoring and auditing by the Sponsor and inspection by local and regulatory authorities.

12.3 Protocol deviation

Any deviations from the protocol will be documented in a protocol deviation form and filed in the trial master file. Each deviation will be assessed as to its impact on volunteer safety and study conduct. Significant deviations will be listed in the end of study report.

12.4 Audit & inspection

The QA manager conducts systems based internal audits to check that trials are being conducted according to local procedures and in compliance with GCP and applicable regulations.

The Sponsor, trial sites, and ethical committee(s) may carry out audit to ensure compliance with the protocol, GCP and appropriate regulations.

GCP inspections may also be undertaken by the MHRA to ensure compliance with protocol and the Medicines for Human Use (Clinical Trials) Regulations 2004, as amended. The Sponsor will assist in any inspections and will support the response to the MHRA as part of the inspection procedure.

©Oxford University

13. SERIOUS BREACHES

The Medicines for Human Use (Clinical Trials) Regulations contain a requirement for the notification of "serious breaches" to the MHRA within 7 days of the Sponsor becoming aware of the breach.

A serious breach is defined as “A breach of GCP or the trial protocol which is likely to effect to a significant degree

(a) the safety or physical or mental integrity of the subjects of the trial; or

(b) the scientific value of the trial”.

In the event that a serious breach is suspected the Sponsor will be informed within one working day.

©Oxford University

14. ETHICS AND REGULATORY CONSIDERATIONS

14.1 Declaration of Helsinki

The Investigators will ensure that this study is conducted according to the principles of the current revision of the Declaration of Helsinki.

14.2 Guidelines for Good Clinical Practice

The Investigators will ensure that this study is conducted in full conformity with the Good Clinical Practice (GCP).

14.3 Approvals

The protocol, informed consent form, participant information sheet and any proposed advertising material will be submitted to an appropriate Research Ethics Committee (REC), HRA (where required), regulatory authorities (MHRA in the UK), and host institution(s) for written approval.

The Investigator will submit and, where necessary, obtain approval from the above parties for all substantial amendments to the original approved documents.

No substantial amendments to this protocol will be made without consultation with, and agreement of, the Sponsor. Any substantial amendments to the trial that appear necessary during the course of the trial must be discussed by the Investigator and Sponsor concurrently. If agreement is reached concerning the need for an amendment, it will be produced in writing by the Chief Investigator and will be made a formal part of the protocol following ethical and regulatory approval.

The Investigator is responsible for ensuring that changes to an approved trial, during the period for which regulatory and ethical committee(s) approval has already been given, are not initiated without regulatory and ethical committee(s)' review and approval except to eliminate apparent immediate hazards to the subject.

14.4 Volunteer Confidentiality

All data will be anonymised: volunteer data will be identified by a unique study number in the CRF and database. A separate confidential file containing identifiable information will be stored in a secured location in accordance with the relevant data protection legislation. Only the Sponsor representative, Investigators, the clinical monitor, the REC and the MHRA will have access to the records. Photographs taken of vaccination sites (if required, with the volunteer's written, informed consent) will not include the volunteer's face and will be identified by the date, trial code and subject's unique identifier. Once developed, photographs will be stored as confidential records, as above. This material may be shown to other professional staff, used for educational purposes, or included in a scientific publication.

©Oxford University

15. FINANCING AND INSURANCE

15.1 Financing

The study is funded by the UK Biotechnology and Biological Sciences Research Council (BBSRC) and the Medical Research Council (MRC)/Department of Health, through the UK Vaccines Network.

15.2 Insurance

The University has a specialist insurance policy in place, which would operate in the event of any participant suffering harm as a result of their involvement in the research (Newline Underwriting Management Ltd, at Lloyd's of London).

15.3 Compensation

Volunteers will be compensated for their time and for the inconvenience caused by procedures. They will be compensated £25 for attending the screening visit. For all other trial visits as outlined in Table 5, compensation will be calculated according to the following:

- Travel expenses:
  - £15 per visit. Where travel expenses are greater than £15 per visit because the volunteer lives outside the city of the trial site, the volunteer will be given further reimbursement to meet the cost of travel necessary for study visits.

- Inconvenience of blood tests:
  - £10 per blood donation

- Time required for visit:
  - £20 per hour

The total amount compensated will be approximately £370 depending on the exact number of visits and whether any repeat or additional visits are necessary.

16. CONTRACTUAL ARRANGEMENTS

An appropriate service level agreement will be put in place between the Sponsor and Oxford University Hospitals NHS Foundation Trust to cover arrangements for processing of blood samples.

©Oxford University

17. PUBLICATION POLICY

The Investigators will be involved in reviewing drafts of the manuscripts, abstracts, press releases and any other publications arising from the study. Data from the study may also be used as part of a thesis for a PhD or MD.

18. DEVELOPMENT OF A NEW PRODUCT/PROCESS OR THE GENERATION OF INTELLECTUAL PROPERTY

Ownership of IP generated by employees of the University vests in the University. The protection and exploitation of any new IP is managed by the University's technology transfer office, Oxford University Innovations.

©Oxford University

19. REFERENCES

1. Linthicum KJ, Britch SC, Anyamba A. Rift Valley Fever: An Emerging Mosquito-Borne Disease. Annual review of entomology. 2016;61:395-415.

2. Easterday BC. Rift valley fever. Advances in veterinary science. 1965;10:65-127.

3. Andriamandimby SF, Randrianarivo-Solofoniaina AE, Jeanmaire EM, Ravololomanana L, Razafimanantsoa LT, Rakotojoelinandrasana T, et al. Rift Valley fever during rainy seasons, Madagascar, 2008 and 2009. Emerging infectious diseases. 2010;16(6):963-70.

4. Madani TA, Al-Mazrou YY, Al-Jeffri MH, Mishkhas AA, Al-Rabeah AM, Turkistani AM, et al. Rift Valley fever epidemic in Saudi Arabia: epidemiological, clinical, and laboratory characteristics. Clinical infectious diseases : an official publication of the Infectious Diseases Society of America. 2003;37(8):1084-92.

5. Daubney R, Hudson JR, Garnham PC. Enzootic hepatitis or rift valley fever. An undescribed virus disease of sheep cattle and man from east africa. The Journal of Pathology and Bacteriology. 1931;34(4):545-79.

6. Nicholas DE, Jacobsen KH, Waters NM. Risk factors associated with human Rift Valley fever infection: systematic review and meta-analysis. Tropical medicine & international health : TM & IH. 2014;19(12):1420-9.

7. Mohamed M, Mosha F, Mghamba J, Zaki SR, Shieh WJ, Paweska J, et al. Epidemiologic and clinical aspects of a Rift Valley fever outbreak in humans in Tanzania, 2007. The American journal of tropical medicine and hygiene. 2010;83(2 Suppl):22-7.

8. Al-Hazmi M, Ayoola EA, Abdurahman M, Banzal S, Ashraf J, El-Bushra A, et al. Epidemic Rift Valley fever in Saudi Arabia: a clinical study of severe illness in humans. Clinical infectious diseases : an official publication of the Infectious Diseases Society of America. 2003;36(3):245-52.

9. Anyamba A, Chretien J-P, Small J, Tucker CJ, Formenty PB, Richardson JH, et al. Prediction of a Rift Valley fever outbreak. Proceedings of the National Academy of Sciences of the United States of America. 2009;106(3):955-9.

10. Bird BH, Nichol ST. Breaking the chain: Rift Valley fever virus control via livestock vaccination. Current opinion in virology. 2012;2(3):315-23.

11. Baba M, Masiga DK, Sang R, Villinger J. Has Rift Valley fever virus evolved with increasing severity in human populations in East Africa? Emerging Microbes & Infections. 2016;5(6):e58.

12. WHO. Fact Sheet: Rift Valley Fever: World Health Organization; 2017 [updated July 2017. Available from: http://www.who.int/mediacentre/factsheets/fs207/en/.

13. Sumaye RD, Geubbels E, Mbeyela E, Berkvens D. Inter-epidemic transmission of Rift Valley fever in livestock in the Kilombero River Valley, Tanzania: a cross-sectional survey. PLoS Neglected Tropical Diseases. 2013;7(8):e2356.

14. Mhina AD. Inter–epidemic transmission of rift valley fever virus in Ngorongoro District, Northern Tanzania: Sokoine University of Agriculture; 2016.

15. Henderson B, McCrae A, Kirya B, Ssenkubuge Y, Sempala S. Arbovirus epizootics involving man, mosquitoes and vertebrates at Lunyo, Uganda 1968. Annals of Tropical Medicine & Parasitology. 1972;66(3):343-55.

16. UMo. H. Press statement on outbreak of Rift Valley Fever in the country. 2016.

17. Ministry of Health U. Rift Valley Fever (RVF) Outbreak Confirmed in Mityana and Kiboga Districts. Press Release 21 Nov 2017. 2017.

18. Magona J, Galiwango T, Walubengo J, Mukiibi G. Rift Valley fever in Uganda: seroprevalence and risk factor surveillance vis-à-vis mosquito vectors, anti-RVF virus IgG and RVF virus neutralizing antibodies in goats. Small ruminant research. 2013;114(1):176-81.

©Oxford University

19. Rolin AI, Berrang-Ford L, Kulkarni MA. The risk of Rift Valley fever virus introduction and establishment in the United States and European Union. Emerg Microbes Infect. 2013;2(12):e81.

20. Warimwe GM, Gesharisha J, Carr BV, Otieno S, Otingah K, Wright D, et al. Chimpanzee Adenovirus Vaccine Provides Multispecies Protection against Rift Valley Fever. Scientific reports. 2016;6:20617.

21. Dungu B, Donadeu M, Bouloy M. Vaccination for the control of Rift Valley fever in enzootic and epizootic situations. Vaccines and Diagnostics for Transboundary Animal Diseases. 135: Karger Publishers; 2013. p. 61-72.

22. Smithburn K. Rift Valley fever: the neurotropic adaptation of the virus and the experimental use of this modified virus as a vaccine. British journal of experimental pathology. 1949;30(1):1.

23. Pittman PR, Liu C, Cannon TL, Makuch RS, Mangiafico JA, Gibbs PH, et al. Immunogenicity of an inactivated Rift Valley fever vaccine in humans: a 12-year experience. Vaccine. 1999;18(1):181-9.

24. Rusnak JM, Gibbs P, Boudreau E, Clizbe DP, Pittman P. Immunogenicity and safety of an inactivated Rift Valley fever vaccine in a 19-year study. Vaccine. 2011;29(17):3222-9.

25. Kortekaas J. One Health approach to Rift Valley fever vaccine development. Antiviral research. 2014;106:24-32.

26. Bird BH, Khristova ML, Rollin PE, Ksiazek TG, Nichol ST. Complete genome analysis of 33 ecologically and biologically diverse Rift Valley fever virus strains reveals widespread virus movement and low genetic diversity due to recent common ancestry. Journal of virology. 2007;81(6):2805-16.

27. Smithburn KC, Mahaffy AF, et al. Rift Valley fever; accidental infections among laboratory workers. Journal of immunology (Baltimore, Md : 1950). 1949;62(2):213-27.

28. Spiegel M, Plegge T, Pöhlmann S. The Role of Phlebovirus Glycoproteins in Viral Entry, Assembly and Release. Viruses. 2016;8(7):202.

29. Wichgers Schreur PJ, Oreshkova N, Harders F, Bossers A, Moormann RJ, Kortekaas J. Paramyxovirus-based production of Rift Valley fever virus replicon particles. The Journal of general virology. 2014;95(Pt 12):2638-48.

30. Bett AJ, Haddara W, Prevec L, Graham FL. An efficient and flexible system for construction of adenovirus vectors with insertions or deletions in early regions 1 and 3. Proceedings of the National Academy of Sciences of the United States of America. 1994;91(19):8802-6.

31. Top FH, Jr., Grossman RA, Bartelloni PJ, Segal HE, Dudding BA, Russell PK, et al. Immunization with live types 7 and 4 adenovirus vaccines. I. Safety, infectivity, antigenicity, and potency of adenovirus type 7 vaccine in humans. J Infect Dis. 1971;124(2):148-54.

32. Baden LR, Walsh SR, Seaman MS, Tucker RP, Krause KH, Patel A, et al. First-in-human evaluation of the safety and immunogenicity of a recombinant adenovirus serotype 26 HIV-1 Env vaccine (IPCAVD 001). The Journal of infectious diseases. 2012;207(2):240-7.

33. Radošević K, Rodriguez A, Lemckert AA, van der Meer M, Gillissen G, Warnar C, et al. The Th1 immune response to Plasmodium falciparum circumsporozoite protein is boosted by adenovirus vectors 35 and 26 with a homologous insert. Clinical and Vaccine Immunology. 2010;17(11):1687-94.

34. Abel B, Tameris M, Mansoor N, Gelderbloem S, Hughes J, Abrahams D, et al. The novel tuberculosis vaccine, AERAS-402, induces robust and polyfunctional CD4+ and CD8+ T cells in adults. American journal of respiratory and critical care medicine. 2010;181(12):1407-17.

35. Barnes E, Folgori A, Capone S, Swadling L, Aston S, Kurioka A, et al. Novel adenovirus-based vaccines induce broad and sustained T cell responses to HCV in man. Science translational medicine. 2012;4(115):115ra1-ra1.

©Oxford University

36. Milligan ID, Gibani MM, Sewell R, Clutterbuck EA, Campbell D, Plested E, et al. Safety and immunogenicity of novel adenovirus type 26–and modified vaccinia Ankara–vectored Ebola vaccines: a randomized clinical trial. Jama. 2016;315(15):1610-23.

37. Grabenstein JD, Pittman PR, Greenwood JT, Engler RJ. Immunization to protect the US Armed Forces: heritage, current practice, and prospects. Epidemiol Rev. 2006;28:3-26.

38. Xiang Z, Li Y, Cun A, Yang W, Ellenberg S, Switzer WM, et al. Chimpanzee adenovirus antibodies in humans, sub-Saharan Africa. Emerging infectious diseases. 2006;12(10):1596.

39. Farina SF, Gao GP, Xiang ZQ, Rux JJ, Burnett RM, Alvira MR, et al. Replication-defective vector based on a chimpanzee adenovirus. J Virol. 2001;75(23):11603-13.

40. Bruna-Romero O, Gonzalez-Aseguinolaza G, Hafalla JC, Tsuji M, Nussenzweig RS. Complete, long-lasting protection against malaria of mice primed and boosted with two distinct viral vectors expressing the same plasmodial antigen. Proceedings of the National Academy of Sciences of the United States of America. 2001;98(20):11491-6.

41. Tatsis N, Tesema L, Robinson ER, Giles-Davis W, McCoy K, Gao GP, et al. Chimpanzee-origin adenovirus vectors as vaccine carriers. Gene Ther. 2006;13(5):421-9.

42. Ledgerwood JE, DeZure AD, Stanley DA, Coates EE, Novik L, Enama ME, et al. Chimpanzee adenovirus vector Ebola vaccine. New England Journal of Medicine. 2017;376(10):928-38.

43. Sheehy SH, Duncan CJ, Elias SC, Collins KA, Ewer KJ, Spencer AJ, et al. Phase Ia clinical evaluation of the Plasmodium falciparum blood-stage antigen MSP1 in ChAd63 and MVA vaccine vectors. Molecular Therapy. 2011;19(12):2269-76.

44. Swadling L, Capone S, Antrobus RD, Brown A, Richardson R, Newell EW, et al. A human vaccine strategy based on chimpanzee adenoviral and MVA vectors that primes, boosts, and sustains functional HCV-specific T cell memory. Science translational medicine. 2014;6(261):261ra153-261ra153.

45. Antrobus RD, Coughlan L, Berthoud TK, Dicks MD, Hill AV, Lambe T, et al. Clinical assessment of a novel recombinant simian adenovirus ChAdOx1 as a vectored vaccine expressing conserved Influenza A antigens. Molecular Therapy. 2014;22(3):668-74.

46. Rampling T, Ewer K, Bowyer G, Wright D, Imoukhuede EB, Payne R, et al. A monovalent chimpanzee adenovirus Ebola vaccine—preliminary report. New England Journal of Medicine. 2015:150202093719007-.

47. Green CA, Scarselli E, Sande CJ, Thompson AJ, de Lara CM, Taylor KS, et al. Chimpanzee adenovirus–and MVA-vectored respiratory syncytial virus vaccine is safe and immunogenic in adults. Science translational medicine. 2015;7(300):300ra126-300ra126.

48. Dicks MD, Spencer AJ, Edwards NJ, Wadell G, Bojang K, Gilbert SC, et al. A novel chimpanzee adenovirus vector with low human seroprevalence: improved systems for vector derivation and comparative immunogenicity. PLoS One. 2012;7(7):e40385.

49. Warimwe GM, Lorenzo G, Lopez-Gil E, Reyes-Sandoval A, Cottingham MG, Spencer AJ, et al. Immunogenicity and efficacy of a chimpanzee adenovirus-vectored Rift Valley fever vaccine in mice. Virology journal. 2013;10:349.

50. Warimwe GM, Gesharisha J, Carr BV, Otieno S, Otingah K, Wright D, et al. Chimpanzee adenovirus vaccine provides multispecies protection against Rift Valley fever. Scientific reports. 2016;6.

51. Dulal P, Wright D, Ashfield R, Hill AV, Charleston B, Warimwe GM. Potency of a thermostabilised chimpanzee adenovirus Rift Valley Fever vaccine in cattle. Vaccine. 2016;34(20):2296-8.

52. Warimwe GM, Lorenzo G, Lopez-Gil E, Reyes-Sandoval A, Cottingham MG, Spencer AJ, et al. Immunogenicity and efficacy of a chimpanzee adenovirus-vectored Rift Valley fever vaccine in mice. Virology journal. 2013;10(1):349.

©Oxford University

53. Folegatti PM, Ewer KJ, Aley PK, Angus B, Becker S, Belij-Rammerstorfer S, et al. Safety and immunogenicity of the ChAdOx1 nCoV-19 vaccine against SARS-CoV-2: a preliminary report of a phase 1/2, single-blind, randomised controlled trial. The Lancet. 2020;396(10249):467-78.

54. Himeidan YE, Kweka EJ, Mahgoub MM, El Rayah el A, Ouma JO. Recent outbreaks of rift valley Fever in East Africa and the middle East. Frontiers in public health. 2014;2:169.

55. Baudin M, Jumaa AM, Jomma HJ, Karsany MS, Bucht G, Naslund J, et al. Association of Rift Valley fever virus infection with miscarriage in Sudanese women: a cross-sectional study. The Lancet Global health. 2016;4(11):e864-e71.

56. Ewer KJ, O'Hara GA, Duncan CJ, Collins KA, Sheehy SH, Reyes-Sandoval A, et al. Protective CD8+ T-cell immunity to human malaria induced by chimpanzee adenovirus-MVA immunisation. Nature communications. 2013;4:2836.

57. Borthwick N, Ahmed T, Ondondo B, Hayes P, Rose A, Ebrahimsa U, et al. Vaccine-elicited human T cells recognizing conserved protein regions inhibit HIV-1. Molecular therapy : the journal of the American Society of Gene Therapy. 2014;22(2):464-75.

58. Antrobus RD, Coughlan L, Berthoud TK, Dicks MD, Hill AV, Lambe T, et al. Clinical assessment of a novel recombinant simian adenovirus ChAdOx1 as a vectored vaccine expressing conserved Influenza A antigens. Mol Ther. 2014;22(3):668-74.

59. Swadling L, Capone S, Antrobus RD, Brown A, Richardson R, Newell EW, et al. A human vaccine strategy based on chimpanzee adenoviral and MVA vectors that primes, boosts, and sustains functional HCV-specific T cell memory. Sci Transl Med. 2014;6(261):261ra153.

60. Green CA, Scarselli E, Sande CJ, Thompson AJ, de Lara CM, Taylor KS, et al. Chimpanzee adenovirus- and MVA-vectored respiratory syncytial virus vaccine is safe and immunogenic in adults. Science translational medicine. 2015;7(300):300ra126.

61. Ewer K, Rampling T, Venkatraman N, Bowyer G, Wright D, Lambe T, et al. A Monovalent Chimpanzee Adenovirus Ebola Vaccine Boosted with MVA. The New England journal of medicine. 2016;374(17):1635-46.

62. MHRA. Medicines and Healthcare products Regulatory Agency: Coronavirus (COVID-19) vaccine adverse reactions. 22 April 2021.

63. EMA. European Medicines Agency: Direct healthcare professional communication (DHPC): COVID-19 Vaccine Janssen: link between the vaccine and the occurrence of thrombosis in combination with thrombocytopenia. 26 April 2021.

64. Teva Pharmaceuticals USA. Adenovirus Type 4 and Type 7 Vaccine, Live, [package insert]. U.S. Food and Drug Administration website.https://www.accessdata.fda.gov/spl/data/3048ba4b-c546-4c8a-8a37-c8fd2807332c/3048ba4b-c546-4c8a-8a37-c8fd2807332c.xml. Revised April 2020 Accessed 27 April 2021.

65. Choudhry A, Mathena J, Albano JD, Yacovone M, Collins L. Safety evaluation of adenovirus type 4 and type 7 vaccine live, oral in military recruits. Vaccine. 2016;34(38):4558-64.

66. McNeil MM, Paradowska-Stankiewicz I, Miller ER, Marquez PL, Seshadri S, Collins LC, et al. Adverse events following adenovirus type 4 and type 7 vaccine, live, oral in the Vaccine Adverse Event Reporting System (VAERS), United States, October 2011–July 2018. Vaccine. 2019;37(44):6760-7.

67. EMA. European Medicines Agency: COVID-19 vaccine safety update: VAXZEVRIA AstraZeneca AB. 29 March 2021.

68. Voysey M, Clemens SAC, Madhi SA, Weckx LY, Folegatti PM, Aley PK, et al. Safety and efficacy of the ChAdOx1 nCoV-19 vaccine (AZD1222) against SARS-CoV-2: an interim analysis of four randomised controlled trials in Brazil, South Africa, and the UK. The Lancet. 2021;397(10269):99-111.

©Oxford University

69. Emary KR, Golubchik T, Aley PK, Ariani CV, Angus B, Bibi S, et al. Efficacy of ChAdOx1 nCoV-19 (AZD1222) vaccine against SARS-CoV-2 variant of concern 202012/01 (B. 1.1. 7): an exploratory analysis of a randomised controlled trial. The Lancet. 2021.

70. Baden LR, Karita E, Mutua G, Bekker L-G, Gray G, Page-Shipp L, et al. Assessment of the Safety and Immunogenicity of 2 Novel Vaccine Platforms for HIV-1 Prevention: A Randomized Trial. Ann Intern Med. 2016;164(5):313-22.

71. Baden LR, Walsh SR, Seaman MS, Tucker RP, Krause KH, Patel A, et al. First-in-human evaluation of the safety and immunogenicity of a recombinant adenovirus serotype 26 HIV-1 Env vaccine (IPCAVD 001). The Journal of infectious diseases. 2013;207(2):240-7.

72. Barouch DH, Tomaka FL, Wegmann F, Stieh DJ, Alter G, Robb ML, et al. Evaluation of a mosaic HIV-1 vaccine in a multicentre, randomised, double-blind, placebo-controlled, phase 1/2a clinical trial (APPROACH) and in rhesus monkeys (NHP 13-19). Lancet. 2018;392(10143):232-43.

73. Capone S, Brown A, Hartnell F, Sorbo MD, Traboni C, Vassilev V, et al. Optimising T cell (re)boosting strategies for adenoviral and modified vaccinia Ankara vaccine regimens in humans. NPJ vaccines. 2020;5:94.

74. Wilkie M, Satti I, Minhinnick A, Harris S, Riste M, Ramon RL, et al. A phase I trial evaluating the safety and immunogenicity of a candidate tuberculosis vaccination regimen, ChAdOx1 85A prime - MVA85A boost in healthy UK adults. Vaccine. 2020;38(4):779-89.

75. Clinical guidance for healthcare professionals on maintaining immunisation programmes during COVID-19. Public Health England 2020 Publications approval reference: 001559

©Oxford University

Appendix A: Oxford Laboratory Adverse Event Severity Grading

**(Ox) Routine Haematology**			Lab Range	Grade 0	Grade 1	Grade 2	Grade 3
Haemoglobin	**Male**	g/l	130 - 170	126 - 170	115 - 125	100 - 114	<100
	**Female**		120 - 150	114 - 150	105 - 113	90 - 104	<90
White Blood Cells	**Elevated**	x109/l	4.00 - 11.00	3.51 - 11.49	11.50 - 15.00	15.01 - 20.00	>20
	**Low**				2.50 - 3.50	1.50 - 2.49	<1.50
Platelets		x109/l	150 - 400	136 - 400	125 - 135	100 - 124	<100
Neutrophils		x109/l	2.0 - 7.0	1.5 - 7.0	1.00 - 1.49	0.50 - 0.99	<0.50
Lymphocytes		x109/l	1.0 - 4.0	1.0 - 4.0	0.75 - 0.99	0.50 - 0.74	<0.50
Eosinophils		x109/l	0.0 - 0.5	0.0 - 0.64	0.65 - 1.50	1.51 - 5.00	>5.00

**Routine Biochemistry**			Lab Range	Grade 0	Grade 1	Grade 2	Grade 3
Sodium	**Elevated**	mmol/l	135 - 145	135 - 146	147 - 148	149 - 150	>150
	**Low**				132 - 134	130 - 131	<130
Potassium	**Elevated**	mmol/l	3.5 - 5.0	3.4 - 5.0	5.1 - 5.2	5.3 - 5.4	>5.4
	**Low**				3.2 - 3.3	3.0 - 3.1	<3.0
Urea		mmol/l	2.5 - 7.4	2.5 - 8.1	8.2 - 8.9	9.0 - 11.0	>11.0
					1.1-1.5*ULN	>1.5-3.0*ULN	>3.0*ULN
Creatinine		µmol/l	49 - 104	49 - 113	114 -156	157 - 312	>312
					1.3-1.5*ULN	>1.5-2.0*ULN	>2.0*ULN
Bilirubin	**Normal LFT**	µmol/l	0 - 21	0 - 26	27 - 31	32-42	>42
					1.1-1.25*ULN	>1.25-1.5*ULN	>1.5-1.75*ULN
Bilirubin	**Abormal LFT**	µmol/l	0 - 21	0 - 22	23 - 26	27 - 31	>31
When accompanied by any increase in LFT					1.25 - 2.5*ULN	>2.5 - 5.0*ULN	>5.0*ULN
ALT		IU/l	10 - 45	10 - 55	*56 - 112*	*113 - 225*	*>225*
					1.1 - 2.0*ULN	>2.0 - 3.0*ULN	>3.0*ULN
Alk Phosphatase		IU/l	30 - 130	30 - 142	*143 - 260*	*261 - 390*	*>390*
Albumin		g/l	32 - 50	32 - 50	28 - 31	25 - 27	<25

**Coagulation Studies**							
					1.10*ULN	>1.10 - 1.20*ULN	>1.2*ULN
Prothrombin time		s	9.0 - 12.0	9.0 - 13.1	13.2	13.3 - 14.4	>14.4
(APTT)					1.10 - 1.20*ULN	>1.20 - 1.40*ULN	>1.4*ULN
Activated Partial Thromboblasin Time		s	20.0 - 30.0	20.0 - 32.9	33.0 - 36.0	36.1 - 42.0	>42.0
Fibrinogen		g/l	1.5 - 4.0	1.5 - 4.0	1.25 - 1.49	1.0 - 1.24	<1.0

**Other**							
AST					1.25 - 2.5*ULN	>2.5 - 5.0*ULN	>5.0*ULN
Aspartate transferase		IU/l	15 - 42	15 - 51	*52 - 105*	*106 -210*	*>210*
GGT					1.25 - 2.5*ULN	>2.5 - 5.0*ULN	>5.0*ULN
Gamma-glytamyl transpeptidase		IU/l	15 - 40	15 - 50	*51 - 100*	*101 - 200*	*>200*

VC027 Appendix (OUH - v3.0 27 Jul 2016)

Severity grading criteria for clinically significant laboratory abnormalities; adapted from FDA guidelines (1) using Oxford University Hospitals NHS Foundation Trust laboratory reference ranges.
